# Phosphorus coordinated Rh single-atom sites on nanodiamond as highly regioselective catalyst for hydroformylation of olefins

**DOI:** 10.1038/s41467-021-25061-0

**Published:** 2021-08-04

**Authors:** Peng Gao, Guanfeng Liang, Tong Ru, Xiaoyan Liu, Haifeng Qi, Aiqin Wang, Fen-Er Chen

**Affiliations:** 1 Engineering Center of Catalysis and Synthesis for Chiral Molecules, Department of Chemistry, Fudan University, Shanghai, China; 2 Shanghai Engineering Center of Industrial Asymmetric Catalysis for Chiral Drugs, Shanghai, China; 3 College of Chemistry, Sichuan University, Chengdu, Sichuan China; 4 State Key Laboratory of Catalysis, Dalian Institute of Chemical Physics, Chinese Academy of Sciences, Dalian, China

**Keywords:** Heterogeneous catalysis, Catalyst synthesis, Sustainability

## Abstract

Single-atom Rh catalysts present superior activity relative to homogeneous catalyst in olefins hydroformylation, yet with limited success in regioselectivity control. In the present work, we develop a phosphorus coordinated Rh_1_ single-atom catalyst with nanodiamond as support. Benefiting from this unique structure, the catalyst exhibits excellent activity and regioselectivity in hydroformylation of arylethylenes with wide substrate generality, i.e., with high conversion (>99%) and high regioselectivity (>90%), which is comparable with the homogeneous counterparts. The coordination interaction between Rh_1_ and surface phosphorus species is clarified by ^31^P solid-state NMR and X-ray absorption spectroscopy (XAS). Rh single atoms are firmly anchored over nanodiamond through Rh-P bonds, guaranteeing good stability in the hydroformation of styrene even after six runs. Finally, by using this catalyst, two kinds of pharmaceutical molecules, Ibuprofen and Fendiline, are synthesized efficiently with high yields, demonstrating a new prospect of single-atom catalyst in pharmaceutical synthesis.

## Abstract (web summary)

Single-atom Rh catalysts present superior activity in olefins hydroformylation, yet with limited success in regioselectivity control. Here the authors develop a Rh1 single-atom catalyst with nanodiamond as support, with which good to excellent regioselectivities to branched aldehydes in hydroformylation of terminal olefins are achieved.

## Introduction

Hydroformylation of olefins affords a significantly important industrial process for aldehydes synthesis^1–5^. Homogeneous Rh catalysts with various phosphorus ligands have been widely used in this process for the superior activity and selectivity^6–12^. Despite intensive research in homogeneous Rh-catalyzed hydroformylation, only a few examples succeeded in industry up to now^13–16^. Catalyst separation is an important issue, and remains challenging in most cases. Compared with homogeneous catalysts, heterogenized homogeneous Rh complex or supported Rh nanoparticles developed for hydroformylation have advantages in stability and catalyst recycling, but usually present much lower activities and/or selectivities^17–22^. Therefore, designing efficient heterogeneous Rh catalysts is highly desirable in this aspect.

**Fig. 1:**
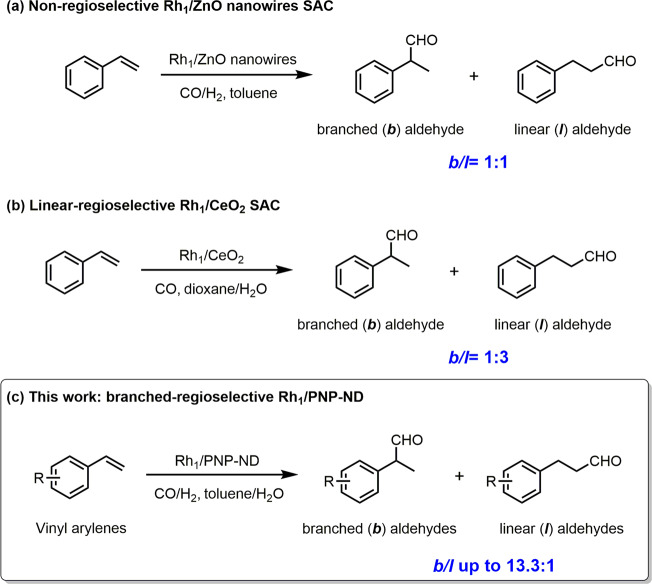
Rh single atom catalysts for regioselective hydroformylation of vinyl arylenes. **a** Rh_1_/ZnO nanowires SAC. **b** Rh_1_/CeO_2_ SAC. **c** the present Rh_1_/PNP-ND catalyst.

Recently, single-atom catalysts (SACs) are emerged as a new type of heterogeneous catalysts, which have demonstrated unexpectedly high specific activity in many reactions^23–32^. The notable characteristic of such kind of catalyst is that active sites are atomically dispersed metal centers on supports. The remarkable reactivity of SACs was related to highly efficient use of metal atoms, and the coordination environment of metal centers with adjacent heteroatoms (O, N, or other metal atoms). The identical coordination environment of isolated metal centers often offered the selectivity comparable to homogeneous counterparts. In the pioneer work in SACs-catalyzed hydroformylation, Zhang et al. demonstrated that Rh_1_/ZnO nanowires SAC showed comparable activity to homogeneous Wilkinson’s catalyst in the hydroformylation of styrene^33^. Although superior activity was obtained, the isolated Rh single atoms on ZnO nanowires failed to control the regioselectivity in this process, which was an important issue in hydroformylation. The ratio of branched and linear aldehydes (*b/l*) maintained at 1:1 when ZnO supported Rh nanocluster was replaced by Rh SAC (Fig. 1a). Recently, the same group devised a Rh_1_/CeO_2_ SAC for the hydroformylation of styrene in the presence of CO, giving a branched/linear aldehyde (*b/l*) ratio of 1:3 (Fig. 1b)^34^. The mechanism investigation revealed that in situ hydrogen was generated via Rh_1_/CeO_2_ SAC-catalyzed water-gas shift reaction, and exhibited a crucial role in regioselectivity. Ding and coworkers designed a series of porous organic copolymers (POPs) through polymerization of various phosphorus ligands, which served as polymer matrix to coordinate with Rh species^35–38^. Such POPs based Rh catalysts presented high activity (TOF > 1200 h^−1^) with excellent regioselectivity to linear aldehydes (*l/b* > 24:1) in the hydroformylation of propene. The authors claimed that partial Rh species were transformed into Rh single-atoms during reaction. Very recently, inorganic phosphorus modified Rh nanoparticles on SiO_2_ was employed in styrene hydroformylation, affording a *b/l* molar ratio of 55:45 with an excellent turnover frequency (1496 h^−1^)^39^. Therefore, combining the advantage of regioselective homogeneous Rh-P complex and efficient heterogeneous catalysts, P-modified Rh SACs might hold great potential in industrial applications. However, the successful preparation of P-coordinated metal single-atom catalyst was rarely reported. Until recently, Li and co-workers reported a graphitic P-coordinated Fe single-atom catalyst, which displayed excellent catalytic performance in hydrogenation and reductive amination reactions for the production of various amines as well as drug targets^40^.

Herein we report the preparation of nanodiamond-supported phosphorus coordinated Rh_1_ species by metal-ligand coordination, abbreviated as Rh_1_/PNP-ND, for aqueous biphasic hydroformylation reactions. In the hydroformylation of styrene, Rh_1_/PNP-ND afforded the highest activity compared to other heterogenized homogenous Rh catalysts and conventional supported Rh catalysts. Rh_1_/PNP-ND exhibited high regioselectivity towards branched aldehyde with a *b/l* molar ratio up to 13.3:1, which outperformed all the supported Rh catalyst in the literature, and was comparable to the homogeneous counterparts (Fig. 1c). Moreover, the original activity and the regioselectivity for the branched α-arylpropionaldehydes still maintained after six runs, suggesting highly stable coordination structure of Rh-P. Besides, various vinyl arenes were converted to the corresponding α-arylpropionaldehydes with remarkable catalytic activity, chemoselectivity, and regioselectivity under mild conditions, indicating the generality of the catalyst. Given the generality and regioselectivity of Rh_1_/PNP-ND, two pharmaceutical molecules Ibuprofen and Fendiline, were synthesized in a step- and atom- economical manner with high yields.

## Results

### Synthesis and characterization of Rh_1_/PNP-ND

**Fig. 2:**
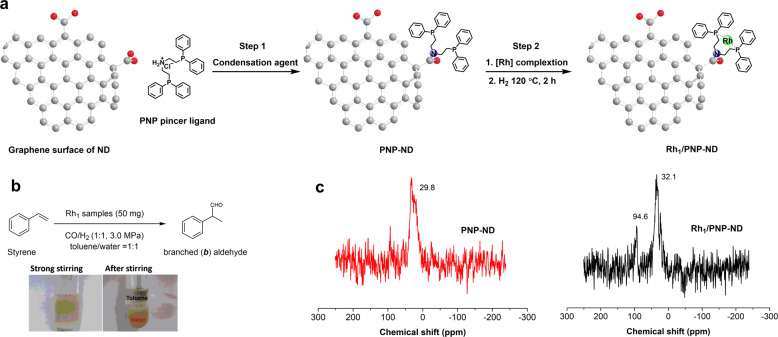
The synthesis of Rh1/PNP-ND. **a** Schematic illustration for synthesizing Rh_1_/PNP-ND. **b** Catalytic hydroformylation of styrene over the synthesized Rh samples. **c** Solid-state ^31^P NMR data.

Pristine and functionalized nanodiamond have been employed as catalyst or support in various catalytic transformations. Structurally, nanodiamond comprised of diamond core (*sp*^3^ carbon) and graphene surface (*sp*^2^ carbon) after annealing at high temperature. It is well known that dispersible ND possessed abundant carboxyl and hydroxyl groups on the graphene surface (further confirmed by XPS analysis in Supplementary Fig. 1b), which facilitate the surface modification with diverse organic modifiers. The synthesis procedure for Rh_1_/PNP-ND by taking advantage of substantial carboxyl groups was illustrated in Fig. 2a (detailed information was shown in Supplementary Notes). To begin with our work, a surface modification method was used to graft PNP pincer ligand bis[2-diphenylphosphinoethyl]amine covalently on ND. In step 1, the surface carboxyl groups selectively reacted with the amino groups of PNP ligands with the catalysis of condensation agents, giving the PNP-ND samples. Subsequently, PNP-ND was impregnated in organic solution of Rh precursor (step 2). Rh species were anchored on PNP-ND via the coordination interaction with surface PNP ligand, and then reduced under hydrogen at 120 °C for 2 h. The catalytic performance of the Rh samples was screened in styrene hydroformylation (Fig. 2b). As shown in Supplementary Tables 1 and 2, the catalytic performances of the synthesized Rh samples were sensitive to the condensation agents, solvents, temperatures and Rh precursors in synthesis process. When NMM (N-methyl morpholine) and CDMT (2-Chloro-4,6-dimethoxy-1,3,5-triazine) was used as condensation agent in step 1, the implantation of [Rh(COD)Cl]_2_ in THF in step 2 gave the most efficient sample (denoted as Rh_1_/PNP-ND except otherwise defined), which showed the highest activity (>99% conversion) with 81% regioselectivity towards branched α-phenylpropionaldehyde (*b/l* = 4.2:1). ^31^P solid-state NMR spectroscopy in Fig. 2c provided the direct evidence of successfully anchoring Rh species on PNP-ND. The ^31^P NMR spectra of PNP-ND presented one peak at 32.1 ppm, corresponding to the PNP ligands immobilized on ND surface. By contrast, Rh_1_/PNP-ND exhibited a major peak for immobilized PNP ligands at 32.1 ppm, and a new peak at 94.6 ppm. The ^31^P chemical shift is a sensitive indicator of the coordination state of PNP pincer ligands^41–43^. The downfield shift of phosphorus resonance was attributed to the coordination of PNP pincer ligands with Rh though the P donor. This evidences PNP ligands served as hosts for Rh anchoring. X-ray photoelectron spectroscopic (XPS) measurements demonstrated the presence of Rh, P and N elements on Rh_1_/PNP-ND (Supplementary Fig. 1). The spectra of P *2p* displayed two peaks at 133.6 and 132.7 eV after deconvolution (Supplementary Fig. 1c). The former peak centered at high binding energy was attributed to P species coordinated with Rh, while the latter one was assigned to uncoordinated PNP. The interpretation of the C1s peak (Supplementary Fig. 1b) revealed three peaks. The peaks at 284.6 and 285.9 eV were assigned to *sp*^2^ and *sp*^3^ carbon atoms respectively. The peak at 287.0 eV represents C=O like carbon atoms (mainly carboxyl groups). The molar ratio of *sp*^2^/*sp*^3^/ C=O like carbon species on the surface was 56:19:25, which revealed an enriched graphene surface with large amounts of carboxyl groups. The binding energy of Rh 3*d*_5/2_ showed two peaks at 309.6 and 308.6 eV, respectively (Supplementary Fig. 1d), which is much higher than that of metallic (306.9 eV) in literature^33^, indicating positively charged Rh state. According to the result of inductively coupled plasma atomic emission spectrometry (ICP-AES), the loading of metal Rh species on PNP-ND is 0.5 wt%.

**Fig. 3:**
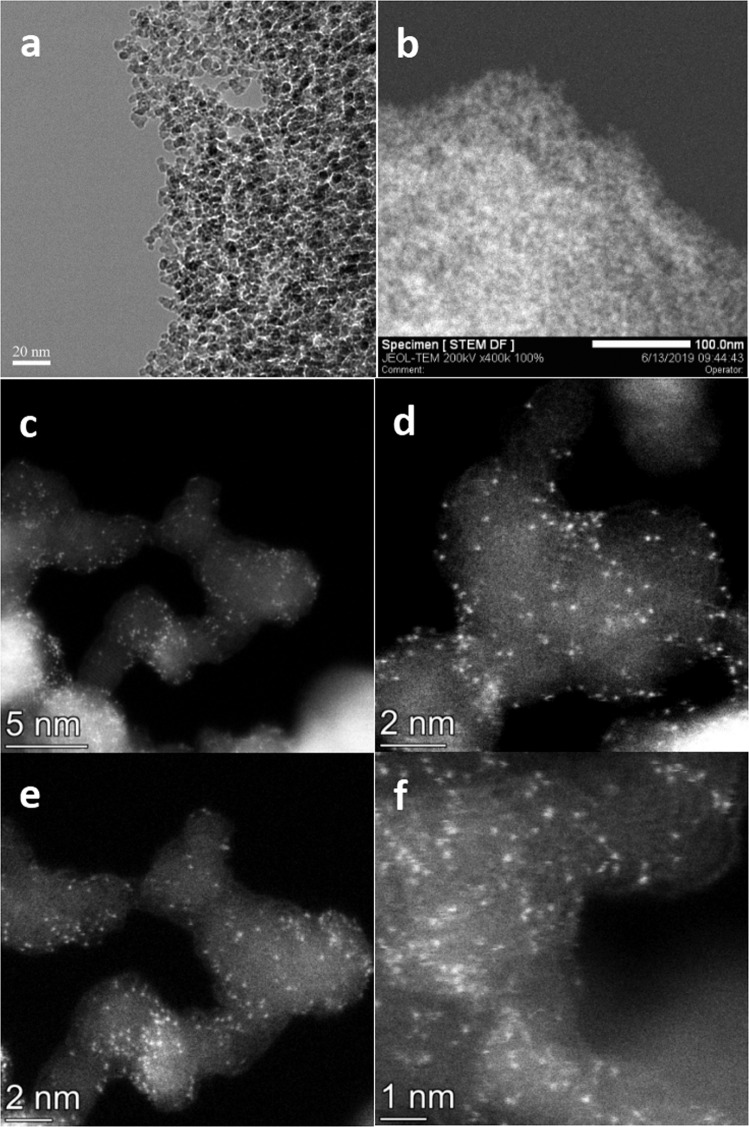
Structure and Morphology characterization of Rh1/PNP-ND. **a** TEM image. **b** HAADF-STEM image. **c**–**f** AC-HAADF-STEM images.

Rh_1_/PNP-ND was further characterized with atomic-resolution characterization techniques. High resolution transmission electron microscopy (HRTEM) revealed that the diameter of ND particles is about 5–10 nm, and no Rh cluster was observed (Fig. 3a). In order to determine the dispersion states of Rh atoms, Rh_1_/PNP-ND was investigated by the aberration-corrected high-angle annular dark-field scanning transmission electron microscopy (AC-HAADF-STEM). As shown in Fig. 3b–f, isolated bright spots were exclusively probed on Rh_1_/PNP-ND, revealing the atomically dispersed Rh atoms.

**Fig. 4:**
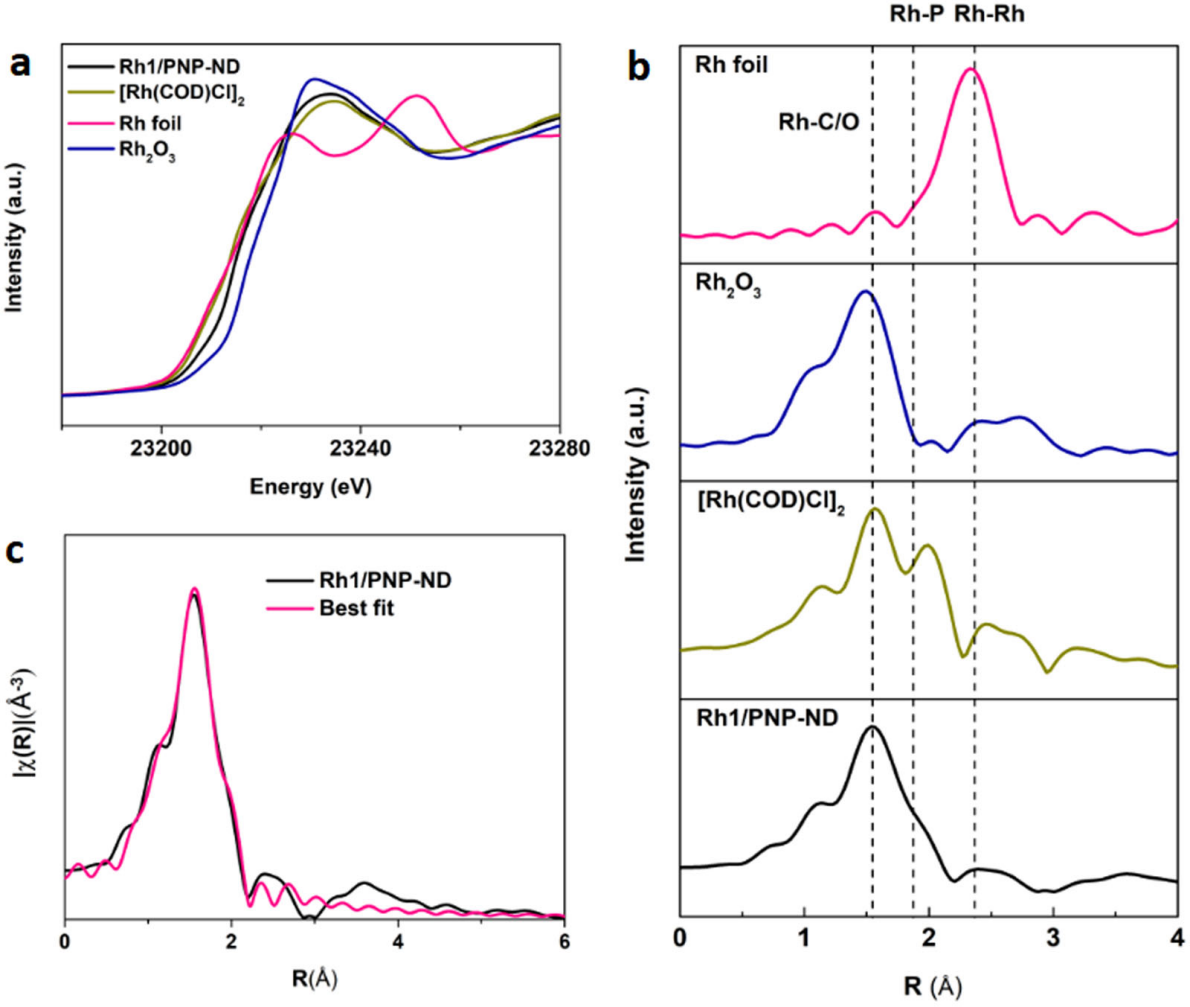
The Rh XAS data of Rh1/PNP-ND and standard Rh materials. **a** The normalized XANES spectra at the Rh K-edge of the Rh_1_/PNP-ND, [Rh(COD)Cl]_2_, Rh_2_O_3_, and Rh foil. **b** The Fourier transform of k^2^-weighted EXAFS spectra at the K-edge of the Rh1/PNP-ND, [Rh(COD)Cl]_2_, Rh_2_O_3_, and Rh foil._._ **c** The experimental Rh EXAFS spectra (black line) and the fitting curve of Rh_1_/PNP-ND (red line).

The X-ray adsorption fine structure (XAFS) was employed to determine the electronic states and the local coordination environment of Rh single atoms on Rh_1_/PNP-ND. The X-ray absorption near-edge structure (XANES) of Rh_1_/PNP-ND clearly showed that Rh species were positively charged (Rh^δ+^, 1 < δ < 3), in comparison with those of [Rh(COD)Cl]_2_ and Rh_2_O_3_ (Fig. 4a). It is in good agreement with the XPS spectra of Rh 3*d*_5/2_ on Rh_1_/PNP-ND. Furthermore, we probed the local coordination environment of isolated Rh atoms by using Fourier-transformed k2-weighted extended X-ray absorption fine structure (EXAFS) in R space. The sample displayed a major scattering peak at 1.5 Å, which was ascribed to the first coordination shell of Rh–C or Rh–O (Fig. 4b). In a related work recently reported by Ma and co-workers, similar results were obtained from the EXAFS spectra of a Cu single-atom catalyst on ND^25^. The authors decisively assigned the peak at 1.5 Å to the first coordination shell of Cu–C. In the present work the contribution of Rh–O scattering was not excluded, considering the positively charged Rh species observed from XPS spectra. The unresolved peak appeared at about 1.9 Å in Fig. 4b, suggesting the first coordination shell of Rh-P. This finding evidences that Rh single atoms anchored on PNP-ND were coordinated with P species, which has been confirmed from the results of ^31^P solid-state NMR and XPS. The best-fitted EXAFS results of Rh_1_/PNP-ND and the corresponding standard samples in the first shell is shown in Fig. 4c and Supplementary Table 3. Rh_1_/PNP-ND showed a Rh–C/O coordination at 2.08 Å with CN of 5.0. The CN of Rh atom with P atoms was 1.6, with a mean bond length of 2.38 Å, which matched approximately with the bidentate chelation of PNP pincer ligands on ND. Based on the above results, the isolated Rh atoms were stabilized by surrounding C/O atoms and P atoms. It is well known that the chelation of phosphine, especially bidentate phosphine, was much stronger than O or C in organometallic complex, herein we proposed that bidentate phosphine of PNP-ND played a predominant role in the coordination chemistry of Rh single atoms.

### Hydroformylation of styrene

In order to understand the superior catalytic performance of Rh_1_/PNP-ND, the pristine Rh-PNP complex and other heterogenized Rh catalysts were prepared and screened in the hydroformylation of styrene in a toluene/water biphasic system with syngas (CO/H_2_ = 1). As shown in Table 1, Under the standard reaction conditions (toluene/H_2_O = 1:1, 3.0 MPa syngas, 50 °C, and 10 h), their catalytic performances were evaluated in terms of activity (TOF), chemoselectivity to aldehydes, and regioselectivity to branched aldehyde. The chemoselectivities for aldehydes over all the tested catalysts were >90%, especially for Rh_1_/PNP-ND, over which >99% selectivity towards aldehydes was obtained. Rh_1_/PNP-ND exhibited 77% conversion (TOF = 95 h^−1^) with the *b/l* ratio up to 12.4:1, which outperformed all the other heterogenized Rh catalysts. Besides Rh_1_/PNP-ND, Rh-PNP/ND was synthesized though physically mixing Rh-PNP complex with unmodified ND by electrostatic adsorption. In comparison, Rh-PNP/ND gave 44% conversion (TOF = 96 h^−1^) with a *b/l* ratio of 4.7:1. Homogeneous Rh-PNP complex gave 85% conversion (TOF = 105 h^−1^) with a *b/l* ratio of 4.9:1. The regioselectivity with Rh-PNP complex is similar to that over Rh-PNP/ND. Unligated [Rh(COD)Cl]_2_/ND prepared by directly immobilizing [Rh(COD)Cl]_2_ on ND, gave much lower activity (17% conversion, and TOF = 27 h^−1^) with inferior regioselectivity (*b/l* = 2.0). A conventional heterogeneous catalyst, ND supported Rh nanoparticles (denoted as Rh NPs/ND), only presented 11% conversion (TOF = 15 h^−1^) with a *b/l* ratio of 1.9:1.

**Table 1:** Screening of various Rh catalysts in the hydroformylation of styrene.

Entry	Catalyst	Rh loading (wt%)	*t* (h)	Conv. (%)	Sel.^a^_*CHO*_ (%)	*b/l*^b^	TOF (h^−1^)
1	Rh_1_/PNP-ND	0.50	10	77	>99	12.4:1	95
2^c^	[Rh(COD)Cl]_2_-PNP	/	10	85	99	4.9:1	105
3	Rh-PNP/ND	0.28	10	44	98	4.7:1	96
4	[Rh(COD)Cl]_2_/ND	0.37	10	17	94	2.0:1	27
5	Rh NPs/ND	0.40	10	11	91	1.9:1	15
6	Rh_1_/PNP-ND	0.50	16	>99	>99	13.1:1	77
7^d^	Rh_1_/PNP-ND	0.50	30	>99	>99	11.9:1	37

Reaction conditions: 3 mmol styrene, 50 mg catalyst, 15 mL toluene, 15 mL H_2_O, 3.0 MPa syngas (CO/H_2_ = 1), 50 °C, The Rh loading was determined by ICP-AES.

^a^The selectivity for aldehydes products.

^b^The molar ratio of branched aldehydes/linear aldehydes.

^c^The molar ratio of Rh/PNP is 1:1, 0.25 mg Rh was used.

^d^200 mg catalyst, 3 mL H_2_O, 12 mmol styrene (1.42 mL).

### Isolated Rh sites with different coordination environment

**Fig. 5:**
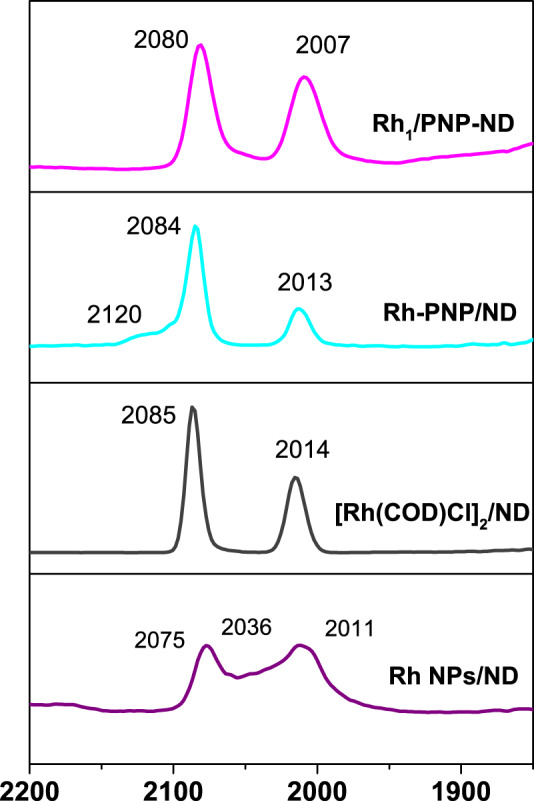
In situ CO-DRIFTs spectra of Rh catalysts. Prior to characterization, all the samples were pretreated under helium at 200 °C for 1 h, and then further treated in 1% CO/He for 1 h. A series of infrared spectra curves for Rh_1_/PNP-ND, Rh-PNP/ND, [Rh(COD)Cl]_2_/ND, and Rh NPs/ND were measured over time (see Supplementary Information for detail).

According to several decades of intensive studies of infrared spectra of chemisorbed CO on Rh, in situ CO-diffuse reflectance infrared Fourier transform spectroscopy (CO-DRIFTs) has been proved to be a powerful technology to distinguish the isolated Rh atoms from other types of Rh species^33,44–48^. The most notable characteristic of isolated Rh species is a well-resolved doublet of chemisorbed CO, which was assigned to symmetric and antisymmetric coupling between *gem*-dicarbonyl Rh(CO)_2_ on isolated Rh species or Rh complex compounds. For Rh_1_/PNP-ND, a well-resolved doublet with components at 2080 and 2007 cm^−1^ was exclusively observed in Fig. 5, indicating the atomically dispersed Rh. In addition, as purging time increased, the position of the doublet for keeps invariant with the decrease in peak intensity (Supplementary Fig. 5). This phenomenon is a distinctive feature of *gem*-dicarbonyl Rh(CO)_2_ on isolated Rh atoms. Similar doublet with components at high wavenumbers (2085 and 2014 cm^−1^) was also observed on [Rh(COD)Cl]_2_/ND (Fig. 5 and Supplementary Fig. 7), suggesting the nature of isolated Rh species. Rh-PNP/ND presented a dominant doublet at 2084 and 2013 cm^−1^ and a very weak prak at 2021 cm^−1^ (Fig. 5 and Supplementary Fig. 6), which clearly revealed that the majority of Rh species existed in the form of isolated sites. For the Rh NPs/ND, a doublet at relatively low wavenumbers (2075 and 2011 cm^−1^), with a overlapped peak centered at 2036 cm^−1^ (Fig. 5 and Supplementary Fig. 8). The former was due to the reduced Rh single atoms, while the later was attributed to the linear CO on metallic Rh cluster. This implied that Rh species on Rh NPs/ND existed in a mixture of isolated Rh atoms and Rh clusters. Such observation is in consistent with the XPS results, which revealed that Rh species on Rh NPs was in metallic states (Supplementary Fig. 4).

The detailed information about CO-DRIFTs was summarized in Supplementary Table 4. According to the wavenumbers of the doublet for dicarbonyl Rh(CO)_2_ in Fig. 5, the electronic density of the isolated Rh species was in an order of Rh_1_/PNP-ND > Rh-PNP/ND > [Rh(COD)Cl]_2_/ND. This trend was further confirmed by XPS spectra of Rh 3*d* in Supplementary Figs. 1–4. The peaks of Rh 3*d*_5/2_ in Rh-PNP/ND, and [Rh(COD)Cl]_2_/ND were centered at 309.9, and 310.0 eV, respectively (Supplementary Figs. 2 and 3), which were higher than those in Rh_1_/PNP-ND (309.8 and 308.7 eV). The higher electron density of Rh single atoms on Rh_1_/PNP-ND was attributed to the obvious electron transfer from PNP ligand to Rh single atom through coordination. From the ratio of integrated absorbance of asymmetric and symmetric stretches, the angle (2ɑ) between carbonyl groups on the isolated Rh atoms was calculated by Paul^’^s formula^45^. The angle (2ɑ) between carbonyl groups on Rh_1_/PNP-ND is calculated to be 104°, while those on Rh-PNP/ND, [Rh(COD)Cl]_2_/ND, Rh NPs/ND, and Rh_1_/ZnO are 120, and 105, 89, and 90°, respectively, suggesting the geometry of Rh(CO)_2_ are closely associated with the coordination environment of the different isolated Rh atoms.

### Origin of regioselectivity

According to the above results, we concluded that the geometry, the electronic states, and the coordination environment of Rh species had crucial effect on the regioselectivity. Rh single atoms on Rh_1_/PNP-ND exhibited relatively high electron density, and big angle between *gem*-dicarbonyl Rh(CO)_2_, which was closely associated with the excellent branched-regioselectivity. The isolated Rh species on Rh-PNP/ND presented bigger angle (2ɑ = 120°) between *gem*-dicarbonyl Rh(CO)_2_, but were more positively charged. As a consequence, Rh-PNP/ND gave a moderate regioselectivity (*b/l* ratio = 4.7:1). The more positively charged isolated Rh species on [Rh(COD)Cl]_2_/ND exhibited even lower regioselectivity (*b/l* ratio of 2.0:1), albeit with a similar angle (2ɑ = 105°) compared to Rh_1_/PNP-ND. Different from the positively charged Rh single atoms on the above three catalysts, non-regioselective Rh_1_/ZnO-nanowires possessing slightly negative charged Rh single atoms, gave a small angle (2ɑ = 94°). This may be responsible for the loss in regioselectivity control. Metallic Rh NPs/ND showed quite inferior regioselectiviy and activity (*b/l* ratio = 1.9:1 with TOF of 15 h^−1^) due to the formation of Rh clusters during hydrogen reduction.

### DFT calculation

**Fig. 6:**
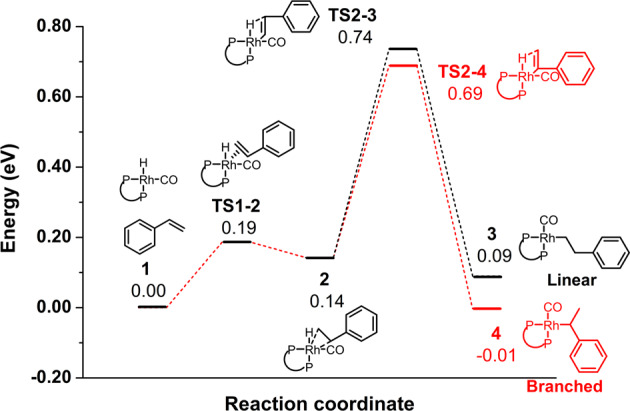
Free energy profiles of the branched, and linear reaction pathways for styrene hydroformylation. All of the calculations were performed with Gaussian09 package (see Supplementary Information for detail). Geometry optimization of all the minima was carried out at the B3LYP level with the 6–31 G* basis set for C, H, N, O, P and Lanl2DZ for Rh. Default convergence criteria were used.

In order to figure out the role of the coordination chemistry of Rh single atoms in Rh_1_/PNP-ND in determining the regioselectivity of the hydroformylation reaction, density functional theory (DFT) calculations were performed. The energy files for the reaction pathways of branched and linear products was illustrated in Fig. 6. The Rh_1_/PNP-ND model was established by coordinating one Rh atom with two P atoms on a graphene-like monolayer (Supplementary Fig. 12). A 4-coordinate square-planar structure with two P atoms **1** was confirmed after optimization. The average Rh-P length in **1** is 2.31 Å, which is in line with EXAFS results (2.38 Å). The barrier of styrene coordination on 5-coordinate Rh single atom **TS1-2** was 0.19 eV. For the linear, and branched reaction pathways, the coordination of styrene were in fact identical. The hydride insertion was crucial in determining the regioselectivity. The reaction barrier of the hydride insertion state for the branched pathway was 0.69 eV, lower than that for the linear pathway (0.74 eV). The reaction energy for the linear-, and branched- alkyl complex was 0.09 and −0.01 eV, respectively. The relative rates for the branched and linear reaction pathways is calculated to be 5.75 and 1.00, which resulted in a predicted ratio of branched to linear selectivity of 85:15. This result matched toughly with the experimental data (in the range of 90:10–95:5), supporting the finding that hydride transfer from Rh single atom to adsorbed styrene played a determining role in the regioselectivity. For the formation of alkyl complex from **2**, ∆G for the branched alkyl complex **4** is −3.36 kcal mol^−1^, while ∆G for the linear one **3** is −1.21 kcal mol^−1^, indicating the branched reaction pathway was also thermodynamically favorable. Therefore, the coordination environment of Rh single atom in Rh_1_/PNP-ND favored the branched reaction pathway thermodynamically and kinetically, which was responsible for the excellent regioselectivity towards the branched aldehydes.

### Stability test

**Fig. 7:**
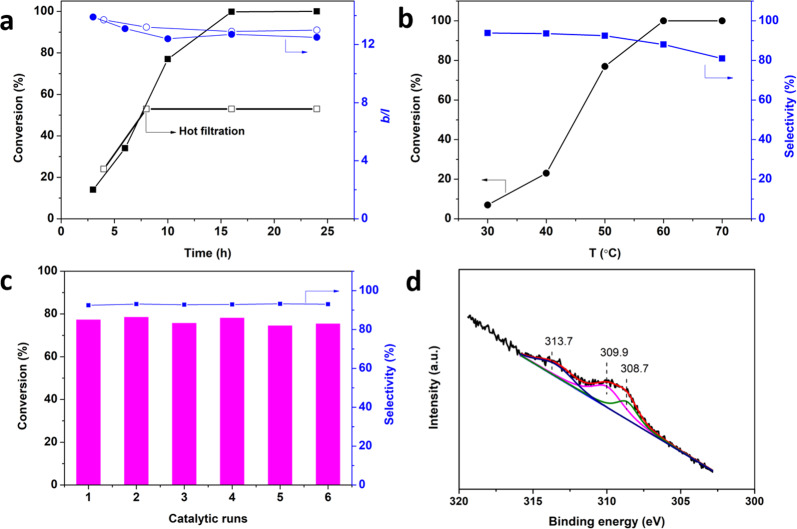
Catalytic performance and characterization of Rh_1_/PNP-ND in stability test. All the experiments were conducted under the reaction conditions of 3 mmol styrene, 50 mg catalyst, 15 mL toluene, 15 mL H_2_O, 3.0 MPa syngas (CO/H_2_ = 1). **a** Time-conversion & regioselectivity curve. Reaction conditions: 50 °C. **b** Effect of reaction temperature on the catalytic performance. Reaction conditions: 10 h. **c** Stability test. Reaction conditions: 50 °C, 10 h. Recovered catalyst was washed with ultra-pure water, centrifuged, and submitted to the next run. **d** XPS survey spectra of Rh 3*d* for the used Rh_1_/PNP-ND after six runs.

The time-conversion/regioselectivity curves in Fig. 7a showed full conversion of styrene was achieved with high regioselectivity (*b/l* > 12) within 16 h. Temperature effect on the activity and regioselectivity was illustrated in Fig. 7b. The regioselectivity decreased with the increase in temperatures. Then the stability of Rh_1_/PNP-ND was investigated in biphasic hydroformylation of styrene. Under a controlled conversion of 77%, Rh_1_/PNP-ND could be recycled at least 6 times without loss in activity and regioselectivity, demonstrating the good stability (Fig. 7c). Furthermore, to rule out the possibility of Rh leaching, hot filtration experiment was conducted at 8 h. No further conversion was observed after removing Rh_1_/PNP-ND, suggesting that the reaction exclusively proceeded heterogeneously. The XPS spectrum of Rh 3*d* show the two peaks at 309.9 and 308.7 eV (Fig. 7d), which was in consistent with those on the fresh catalyst, indicating the electronic states of Rh single atoms were unchanged after recycling. The AC-HAADF-STEM images of the used Rh_1_/PNP-ND reveal that Rh species still existed in the form of single atoms after catalytic runs, and no aggregation or Rh cluster was observed, indicating the robust nature of P-coordinated Rh single atoms (Supplementary Fig. 9). To our delight, Rh_1_/PNP-ND was exclusively dispersed in aqueous phase, after reaction the catalyst was spontaneously separated from the reaction mixtures (Fig. 2b). Benefitting from the hydrophilic nature of Rh_1_/PNP-ND, we conducted the reaction under an organic solvent-free conditions. As shown in Table 1, the present Rh_1_/PNP-ND still displayed high regioslectivity (*b/l* ratio = 11.9:1) with a completed conversion with a prolonged reaction time of 30 h in water.

### Substrate scope

The substrate scope was explored to evaluate the generality of this catalytic system. Table 2 illustrated that vinyl arenes could be transformed to the corresponding aldehydes with excellent chemoselectivities in all cases. Most substituted vinyl arenes exhibited >99% conversion with excellent regioselectivities (>90%), indicating the high efficiency of Rh_1_/PNP-ND. However, sterically crowded 2-fluorostyrene and 2-chlorostyrene gave 68 and 44% conversion within 24 h, respectively, indicating the strong steric hindrance effect of *ortho*- substituents on activity. Further extending reaction time to 60 h afforded full conversion of 2-chlorostyrene with high regioselectivity (*b/l* = 13.1). The electronic effect of the substitutes on regioselectivity was also proven. For instance, *para*-methyl- and *para*-methoxy-substituted vinylarenes afforded relatively low regioselectivities towards the corresponding branched aldehydes, respectively. 2-Vinylnaphthalene was considered as hardly reactive in toluene/water biphase system because of the large steric hindrance and the low water solubility^49–52^. To our delight, 2-vinylnaphthalene was also quite reactive in the present system, giving 98% conversion, high chemoselectivity (>99%) and a *b/l* ratio of 6.2:1 with prolonged time (36 h).

**Table 2:** Rh_1_/PNP-ND catalyzed hydroformylation of substituted vinyl arenes.

Entry	Substrate	Conv. (%)	Sel.^a^_*CHO*_ (%)	*b/l*^b^
1	2-methylstyrene	>99	99	7.3:1
2	3-methylstyrene	>99	99	6.9:1
3	4-methylstyrene	>99	99	6.7:1
4	4-*tert*-butylstyrene	>99	99	8.8:1
5	2,5-dimethylstyrene	>99	99	7.8:1
6	4-methoxystyrene	>99	99	7.5:1
7	3,4-dimethoxystyrene	>99	99	8.1:1
8	2-fluorostyrene	68	99	10.1:1
9	3-fluorostyrene	>99	98	13.3:1
10	4-fluorostyrene	>99	99	10.2:1
11	2-chlorostyrene	44	99	11.5:1
		(>99)^c^	(99)	(13.3:1)
12	3-chlorostyrene	>99	99	15.7:1
13	4-chlorostyrene	>99	99	10.1:1
14	2-bromostyrene	>99^c^	96	13.7:1
15	3-bromostyrene	>99^c^	95	13.6:1
16	4-bromostyrene	>99^c^	98	11.5:1
18	4-hydroxystyrene	>99	99	7.9:1
19^d^	2-vinylnaphthalene	98	99	6.2:1

Reaction conditions: 3 mmol substrate, 50 mg catalyst (Rh loading = 0.5 wt%), 15 mL toluene, 15 mL H_2_O, 3.0 MPa syngas (CO/H_2_ = 1), 50 °C, 24 h.

^a^The selectivity for the aldehydes products.

^b^The molar ratio of branched aldehydes/linear aldehydes.

^c^60 h.

^d^36 h.

For vinyl arenes with insufficient water solubility, the hydroformylation slowly occurred at the water/organic interface and severely limited by mass transport. In the present work, PNP-ND worked as an effective mass transfer promoter for its hydrophobic core and hydrophilic surface. Due to this reason, insoluble vinyl arenes were readily diffused onto the surface of Rh_1_/PNP-ND, and then migrated to the highly dispersible single Rh atom sites. Thus, the reaction rate was greatly accelerated in consequence.

Additionally, terminal aliphatic alkenes (C_6_–C_10_) were also explored to evaluate the regioselectivity of Rh_1_/PNP-ND. It is well known that hydroformylation of terminal aliphatic alkenes usually gave linear aldehydes in high regioselectivity (*b/l* < 0.1), and the branched-selective hydroformylation of terminal aliphatic alkenes was highly challenging. The most recent work was reported by Nozaki and co-workers^53^. With the specifically designed nitrogen-centered triphosphine ligand, the corresponding Rh complex catalysts (1.0 mol% Rh) gave a *b/l* ratio of 1.1 with completed conversion in the hydroformylation of 1-hexene at 100 °C, 3.5 h, and 2.0 MPa. In the present work, Rh_1_/PNP-ND exhibited the *b/l* ratios in the range of 0.58–0.78 (Supplementary Table 5), indicating the branched-selective nature of Rh_1_/PNP-ND.

### Applications in pharmaceutical synthesis

**Fig. 8:**
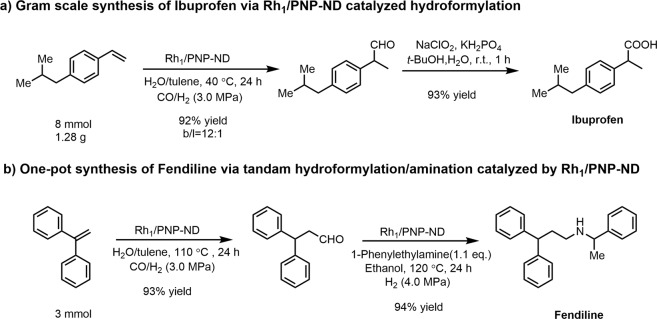
The synthetic routes for Ibuprofen and Fendiline via Rh1/PNP-ND catalyzed hydorformylation. **a** For the hydroformylation step, 200 mg Rh_1_/PNP-ND (Rh loading = 0.5 wt%), 30 mL toluene, 30 mL H_2_O, 40 °C, 24 h, 3.0 MPa CO/H_2_, isolated yield; for the oxidation step, 20 mL *t*-BuOH, 15 mL aq. NaH_2_PO_4_ solution (1.70 M), and 15 mL aq. NaClO_2_ solution (1.0 M), 1 h, isolated yield. **b** for hydroformylation step, 300 mg Rh_1_/PNP-ND (Rh loading = 0.5 wt%), 15 mL toluene, 15 mL H_2_O, 110 °C, 24 h, 3.0 MPa CO/H_2_ (CO/H_2_ = 2), GC yield; for reductive amination step, 300 mg Rh_1_/PNP-ND (Rh loading = 0.5 wt%), 1.1 eq. 1-phenylethylamine, 30 mL ethanol, 120 °C, 24 h, 4.0 MPa H_2_, isolated yield.

The application of heterogeneous single-atom catalysis in pharmaceutical synthesis was rarely reported in the previous literatures^40^. Herein, we conducted two synthetic transformations for preparing two pharmaceutical molecules Ibuprofen and Fendiline, in order to demonstrate the practicability of the present Rh_1_/PNP-ND-catalyzed hydroformylation. As depicted in Fig. 8a, a gram-scale of hydroformylation of 4-isobutylstyrene was efficiently conducted, giving the target branched aldehyde of 92% yield. Then the formed intermediate aldehyde was subjected to mild aqueous oxidation (NaClO_2_, KHPO_2_, 2-methyl-2-butene, *t*-BuOH, H_2_O, 0 °C to r.t., 1 h) in a highly selective manner^54^. Finally, Ibuprofen was obtained in an overall isolated yield of 85%. Moreover, a convenient synthetic route to Fendiline was furnished with one-pot hydroformylation/amination reaction (Fig. 8b). Remarkably, sterically hindered 1,1-diphenylethylene could be converted into the corresponding linear aldehyde in 91% yield, followed by reductive amination with 1-phenylethylamine in the presence of 4.0 MPa H_2_. With this methodology the overall yield of Fendiline was as high as 87%, which was comparable with that using homogeneous rhodium-carbene complexes^55^.

## Discussion

In summary, we have developed a P-coordinated Rh single-atom catalyst (Rh_1_/PNP-ND) by using dispersable nanodiamond as support. Systematic investigation revealed that each isolated Rh atom was firmly anchored on nanodiamond through the bidentate chelation with two P atoms. The electron transfer from the grafted phosphorus ligand to Rh single atom was confirmed by in situ CO-DRIFTs and XPS. This unique structure was analogous to homogeneous Rh-P complex catalysts, and highly related to the remarkable catalytic performance of Rh_1_/PNP-ND in hydroformylation of a series of vinyl arenes. Consequently, diverse α-arylpropionaldehydes, including two important drug intermediates, were produced with excellent yields in a green and efficient way. Our work opened an important prospect of single-atom catalyst in pharmaceutical synthesis.

## Methods

### Synthesis of PNP pincer ligand Bis[2-(diphenylphosphino)ethy]amine

Under nitrogen atmosphere, diphenylphosphine (14.0 mL, 80 mmol) was added to a solution of *tert*-butoxide (14 g, 125 mmol) in anhydrous THF (300 ml) at ambient temperature. And then bis(2-chloroethyl)amine hydrochloride (7.15 g, 40 mmol) was added to the solution. The mixture was stirred for 18 h under reflux. After reaction, the reaction mixture was poured into 400 mL hexane, the organic phase was washed with 10% aqueous NaOH, saturated aqueous NaCl solution, and 2 N aqueous HC1 solution to provide a dense white precipitate of **1·**HCl. Recrystallization from boiling acetonitrile gave a 90% yield (17.2 g) of **1·**HCl of white needles. ^1^H NMR (400 MHz, CDCl_3_): *δ* 2.3-3.3 (m, 8H), 7.0-7.6 (m, 20H), 9.9 (s, 2H).

### Synthesis of Rh_1_/PNP-ND

Nanodiamond (Nanjing XFNANO Materials Tech Co.,Ltd), diphosphino-amine, CDMT (2-Chloro-4,6-dimethoxy-1,3,5-triazine), NMM (N-methyl morpholine), DCC (N,N’-dicyclohexylcarbodiimide), DMAP(4-Dimethylaminopyridine), TEA (triethylamine) were obtained from commercial sources and used without further purification. In step 1, nanodiamond support (50 mg) was dispersed into solvent (12 mL) by ultrasonic treatment for 5 min. And then diphosphino-amine (88 mg, 0.185 mmol), CDMT (32 mg, 0.185 mmol), and NMM (65 mg, 0.65 mmol) were added into the mixture. The slurry was stirred at 90 °C for 48 h under nitrogen atmosphere. After centrifugation, the residual solid washed for three times with DMF and THF, respectively. The sample was dried at 50 °C under vacuum overnight, denoted as PNP-ND.

In step 2, 50 mg PNP-ND and 46 mg [Rh(COD)Cl]_2_ were added in 10 mL solvent, and then stirred at 30 °C for 12 h under N_2_. After that, the mixture was centrifugated, and washed with THF for three times. The solid was dried under vacuum at 50 °C overnight, and then pretreated in hydrogen at 120 °C for 2 h. The obtained sample was denoted as Rh_1_/PNP-ND.

### Catalysts characterization

Solid-state ^31^P NMR spectra were performed on a Bruker 400WB AVANCE III NMR spectrometer with a magnetic field strength of 9.4 T. The metal loading was analyzed by inductively coupled plasma atomic emissionspectroscopy (IRIS Intrepid II XSP, Thermo Electron). STEM characterization was conducted with a JEOL JEM-2100F instrument at 200 kV. The AC-HAADF-STEM images was collected on a JEOL JEM-ARM200F operated at 300 kV, with a guaranteed resolution of 80 pm. The X-ray absorption spectra including X-ray absorption near-edge structure (XANES) and extended X-ray absorption fine structure (EXAFS) at the K-edge of Rh of the samples were collected at the BL 14W1 of Shanghai Synchrotron Radiation Facility (SSRF), China. The Ru foil was employed to calibrate the energy. The spectra were collected under transmission mode at room temperature. The Athena software package was used to analyze the data. In situ CO-diffuse reflectance infrared Fourier transform spectroscopy (DRIFTS) was measured in the range 4000–450 cm^−1^ on a Nicolet 6700 FTIR spectrophotometer (Thermo Fisher). The Rh content in Rh samples and in reaction mixture was determined using an Agilent 730 ICP-OES. X-ray photoelectron spectra (XPS) were performed on an ESCALAB 250 X-ray photoelectron spectrometer equipped with a monochromated Al K*ɑ* anode. ^1^H NMR spectra were recorded at room temperature on 400 MHz Bruker DRX-400 NMR spectrometers.

### Hydroformylaion reaction

The hydroformylation reaction was conducted in a 50 mL high-pressure autoclave. In a typical run, 50 mg catalyst, 3 mmol substrate, 15 mL toluene and 15 mL ultra-pure water were loaded. And then the reactor was purged with syngas (CO/H_2_ = 1), and charged with high-pressure syngas. The reactor was heated to the desired temperature with stirring. After reaction the reaction products were determined by GC/MSD instrument (Agilent 7820A/5977B, equipped with a HP-5 column), and quantified by GC (GC-FID, Agilent 6890A equipped with a DB-624 column).

Experimental procedures for catalyst preparation, characterization, and activity test are provided in Supplementary Information in detail.

## Supplementary information

Supplementary Information

## Data Availability

The data supporting the findings of this study are available from the article and the supplementary information, or from the corresponding authors upon reasonable request.
