# Supplementary material for: Phosphorus coordinated Rh single-atom sites on nanodiamond as highly regioselective catalyst for hydroformylation of olefins
Source: Nat Commun. 2021 Aug 4;12:4698. doi: 10.1038/s41467-021-25061-0 (PMC8339002; doi:10.1038/s41467-021-25061-0)
Supplement: Supplementary file 1 — Supplementary Information [file 41467_2021_25061_MOESM1_ESM.pdf]

Supplementary Information

Phosphorus coordinated Rh single-atom sites on nanodiamond as highly regioselective catalyst for hydroformylation of olefins

Peng Gao[1,2,3], Guanfeng Liang[1,2,✉], Tong Ru[1,2,], Xiaoyan Liu[4], Haifeng Qi[4], Aiqin Wang[4],&

Fen-Er Chen[1,2,3✉]

[1]Engineering Center of Catalysis and Synthesis for Chiral Molecules, Department of Chemistry, Fudan University, No. 220 Handan Road, Shanghai, 200433, China. [2]Shanghai Engineering Center of Industrial Asymmetric Catalysis for Chiral Drugs, Shanghai 200433, China. [3]College of Chemistry, Sichuan University, Chengdu 610064, Sichuan, China. [4]State Key Laboratory of Catalysis, Dalian Institute of Chemical Physics, Chinese Academy of Sciences, 116023 Dalian, China. ✉email: lianggfeng@fudan.edu.cn; rfchen@fudan.edu.cn

Supplementary Table 1. The effect of preparation conditions for nanodiamond supported Rh catalysts on the catalytic performance in styrene hydroformylation.

Entry	Reaction conditions for Step 1	Solvent in Step 2	Conv./%	Sel.^{a}$_{CHO}$/%	*b*/*l*^{b}
1	DCC, DMAP, TEA, THF, 30 °C, 48 h	THF	34.1	98	2.4
2	DCC, DMAP, TEA, DMF, 30 °C, 48 h	DMF	48.3	99	2.9
3	DCC, DMAP, TEA, DMF, 30°C, 48 h	toluene	53.0	98	4.2
4	NMM, CDMT, DMF, 90°C, 48 h	THF	99.5	>99	4.1
5	NMM, CDMT, DMF, 90°C, 48 h	DMF	97.3	>99	2.0
6	NMM, CDMT, DMF, 90°C, 48 h	CH_3CN	90.4	>99	3.1
7	NMM, CDMT, DMF, 90 °C, 48 h	toluene	85.0	>99	3.3
8	NMM, CDMT, DMF, 70 °C, 48 h	THF	31.5	>99	3.6
9	NMM, CDMT, DMF, 130 °C, 48 h	THF	100	>99	2.0

Rection conditions: 50 mg catalyst, 60 °C, 3.0 MPa syngas (CO/H_2=1), 10 h, toluene/H_2O (1:1, 15 mL/15 mL), 3 mmol styrene. **a** The selectivity for aldehyde products. **b** The molar ratio of branched aldehyde/linear aldehyde determined by GC.

Supplementary Table 2. The catalytic performance of Rh samples derived from different Rh precursors.

Entry	Rh precursor	Conv. (%)	Sel.[a]$_{CHO}$ (%)	*b/l*[b]
1	$[Rh(COD)Cl]_2$	99.5	>99	4.1
2	$[Rh(CO)_2(acac)]_2$	99.7	>99	3.8
3	$RhCl_3$	98.2	>99	2.0
4	$Rh(CO)(TPP)_2Cl$	99.7	>99	3.1

Reaction conditions: 50 mg catalyst, 60 °C, 3.0 MPa syngas (CO/H_2=1), 10 h, toluene/H_2O (1:1, 15 mL/15 mL), 3 mmol styrene. **a** The selectivity for aldehyde products. **b** The molar ratio of branched aldehyde/linear aldehyde determined by GC.

Supplementary Table 3. The best-fitted EXAFS results of Rh_1/PNP-ND and the corresponding standard samples.[a]

**Sample**	**Shell**	**CN**	**R(Å)**	**σ^2 (10^{-2} Å^2)**	**ΔE_0 (eV)**	**r-factor (%)**
Rh foil	Rh-Rh	12.0	2.71	0.3	-15.5	0.7
Rh_2O_3	Rh-O	6.0	2.02	0.4	-7.1	0.3
$[Rh(COD)Cl]_2$	Rh-C	4.0	2.11	0.3	-0.1	0.2
	Rh-Cl	2.0	2.41	0.3	-0.1	
Rh_1/PNP-ND	Rh-C/O	5.0	2.08	0.3	-3.7	0.2
	Rh-P	1.6	2.38	0.3	-3.7	

a CN is the coordination number for the absorber-backscatterer pair, R is the average absorber-backscatterer distance, σ^2 is the Debye-Waller factor, and ΔE_0 is the inner potential correction. The accuracies of the above parameters are estimated as CN, ±20%; R, ±1%; σ^2, ± 20%; ΔE_0 , ±20%. The data range used for data fitting in k-space (Δk) and R-space (ΔR) are 2.9-12.8 Å^{-1} and 1.0-2.2 Å, respectively.

Supplementary Table 4. IR bands of chemisorbed CO on various Rh catalysts.

Entry	Rh Catalysts	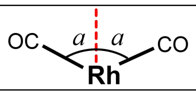		Rh-CO	A_{asym}/A_{sym} [a]	2α (°) [b]
		$\tilde{\nu}_{sym}$ (cm^{-1})	$\tilde{\nu}_{asym}$ (cm^{-1})	$\tilde{\nu}$ (cm^{-1})		
1	Rh_1/PNP-ND	2080	2007	--	1.28	104
2	Rh-PNP/ND	2084	2013	2120 ($Rh^{\delta+}$)	3.03	120
3	$[Rh(COD)Cl]_2$/ND	2085	2014		1.73	105
4	Rh NPs/ND	2075	2011	2036 (Rh^0)	0.99	90
5	Rh_1/ZnO [c]	2087	2014	2060 (Rh^0)	1.14	94

a The ratio of integrated absorbance of asymmetric and symmetric stretches. **b** The value was calculated from the formula $\tan^2\alpha = A_{asym}/A_{sym.}$ c Ref. 1.

Supplementary Table 5. Rh_1/PNP-ND catalyzed hydroformylation of terminal aliphatic alkenes.

Entry	Substrate	Conv. (%)	Sel.[a]$_{CHO}$ (%)	*b/l*[b]
1	1-Hexene	>99	95	0.59:1
2	1-Heptene	>99	93	0.58:1
3	1-Octene	>99	90	0.68:1
4[c]	1-Nonene	>99	82	0.74:1
5[d]	1-Decene	>99	80	0.78:1
6	Cyclohexene	>99[c]	99	/

Reaction conditions: 3 mmol substrate, 50 mg catalyst (Rh loading = 0.5 wt%), 15 mL toluene, 15 mL H_2O, 3.0 MPa syngas (CO/H_2=1), 60 °C, 12 h. **a** The selectivity for the aldehydes products. **b** The molar ratio of branched aldehydes/linear aldehydes. **c** 18 h. **d** 70 °C, 16 h.

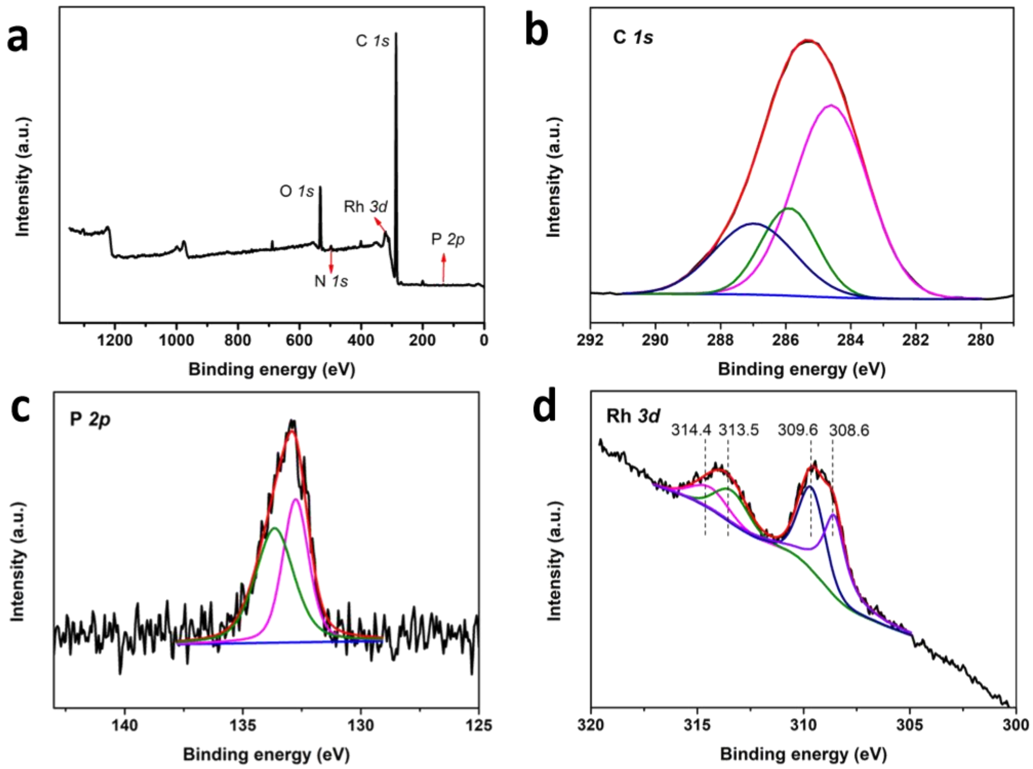

Supplementary Fig. 1. The surface chemical composition and chemical state of Rh_1/PNP-ND. a XPS survey spectra. b C 1*s* spectra. c P 2*p* spectra. d Rh 3*d* spectra.

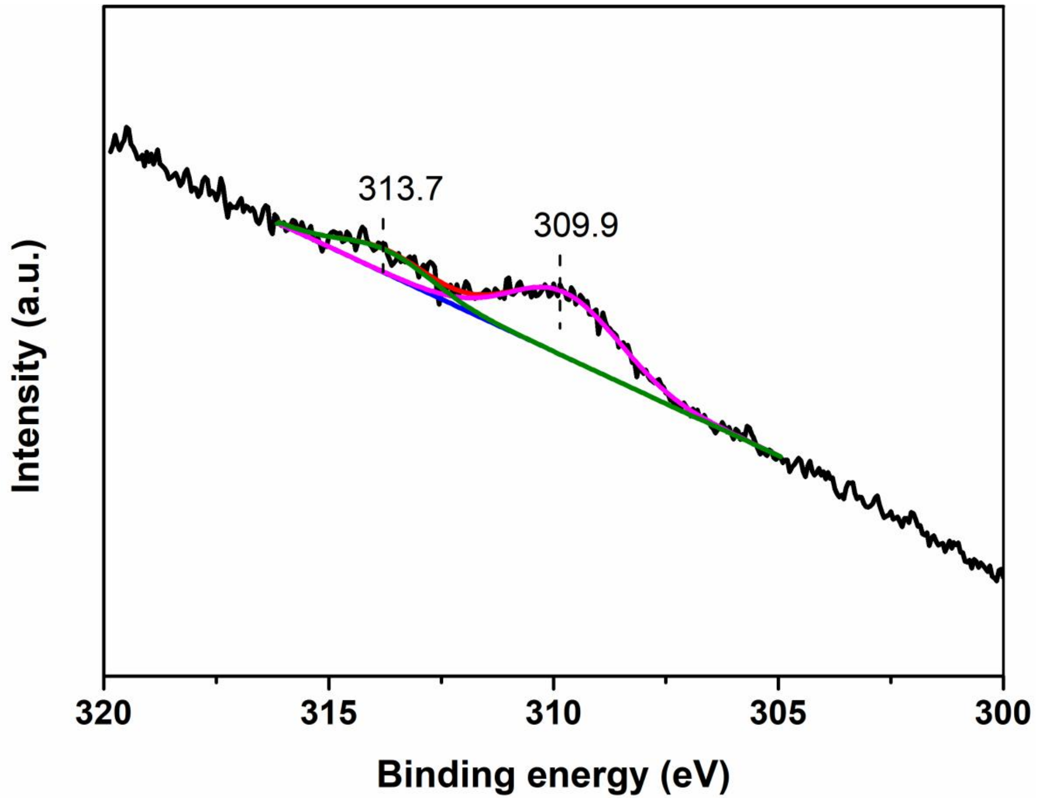

Supplementary Fig. 2. XPS survey spectra of Rh 3*d* on Rh-PNP/ND.

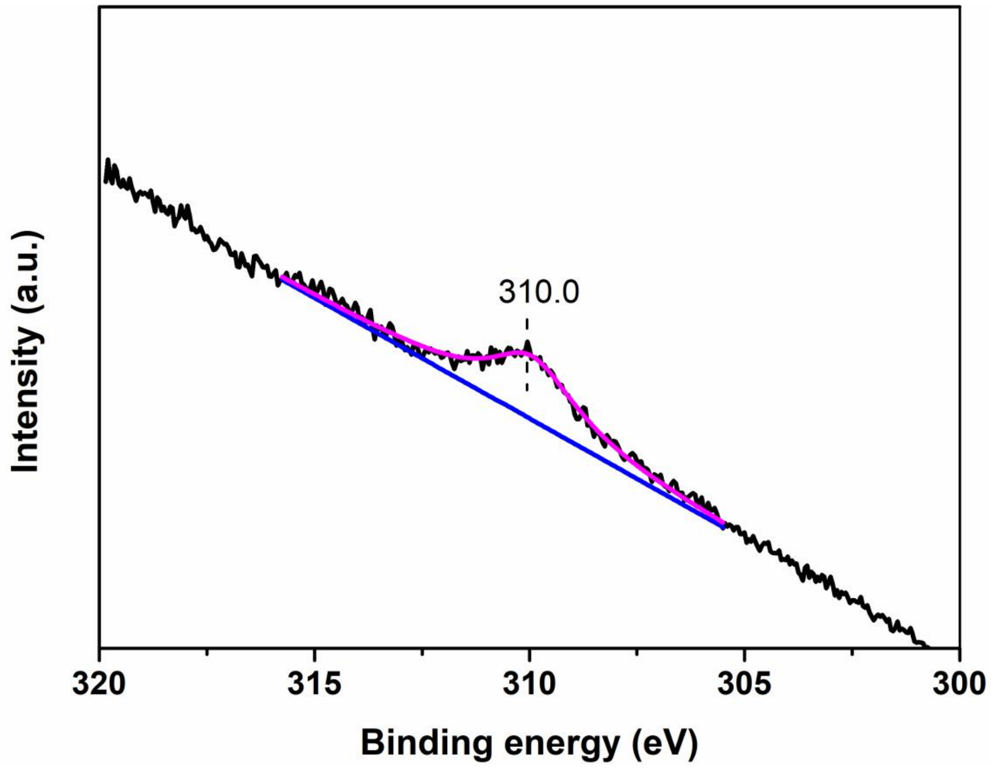

Supplementary Fig. 3. XPS survey spectra of Rh 3*d* on $[Rh(COD)Cl]_2$/ND.

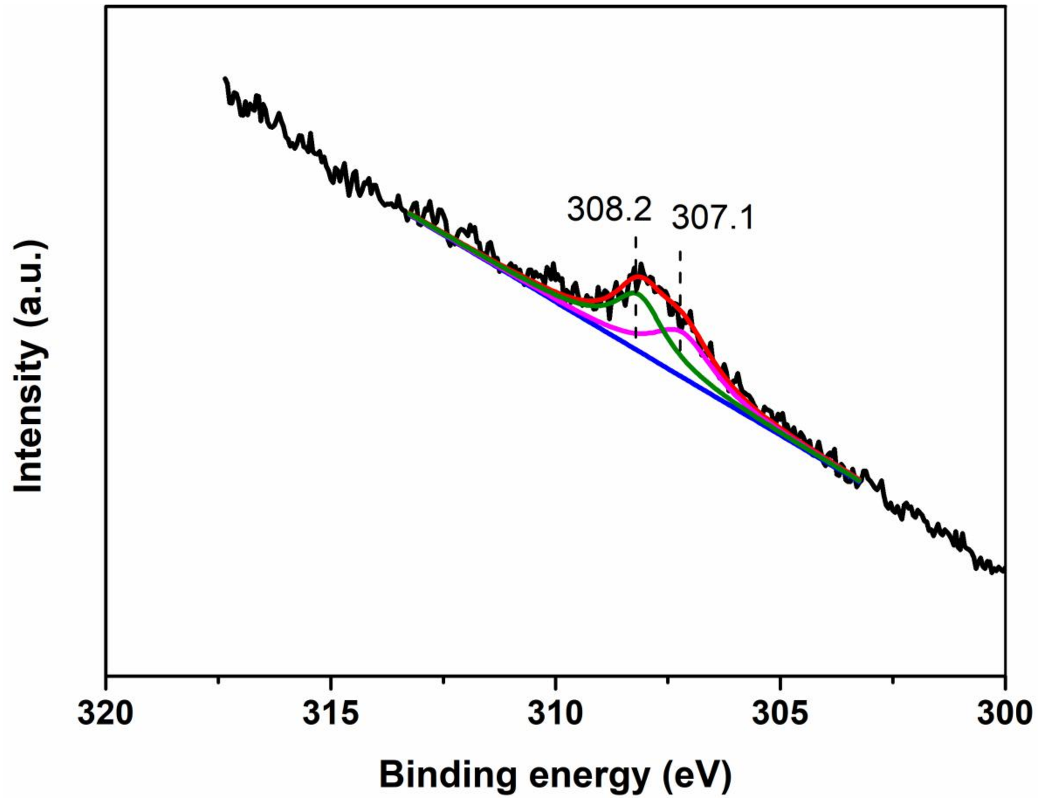

Supplementary Fig. 4. XPS survey spectra of Rh 3*d* on Rh NPs/ND.

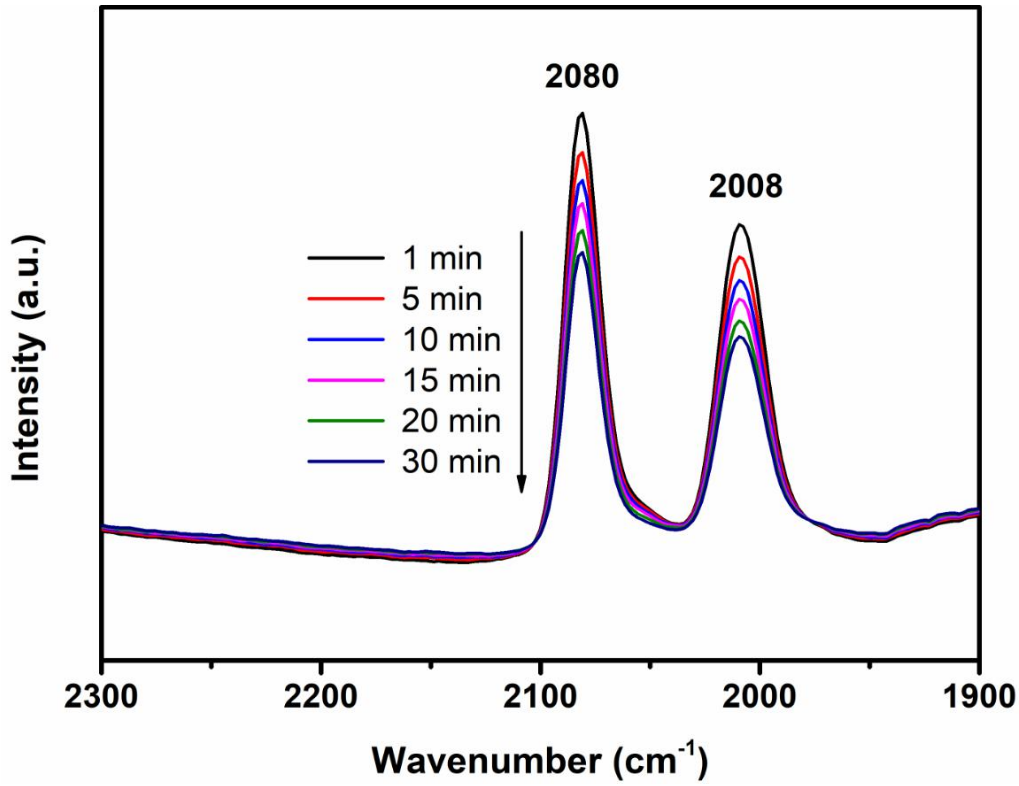

Supplementary Fig. 5. In situ CO-DRIFTs spectra of Rh_1/PNP-ND under He with the different purging time.

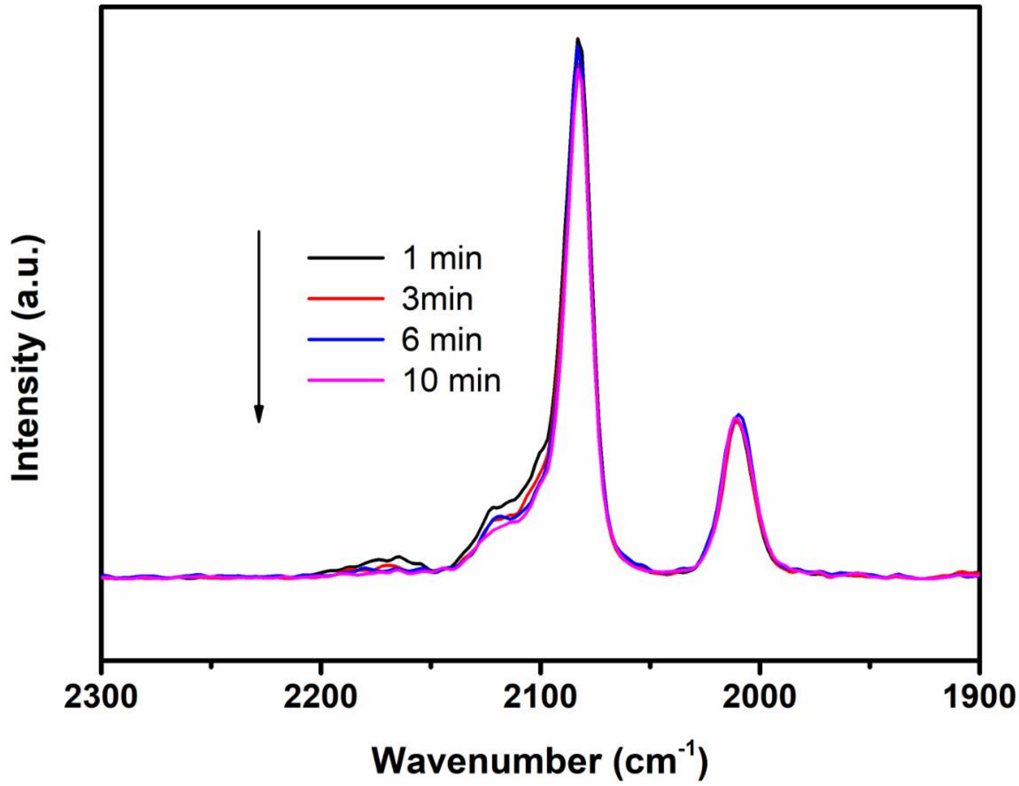

Supplementary Fig. 6. In situ CO-DRIFTs spectra of Rh-PNP/ND under He with the different purging time.

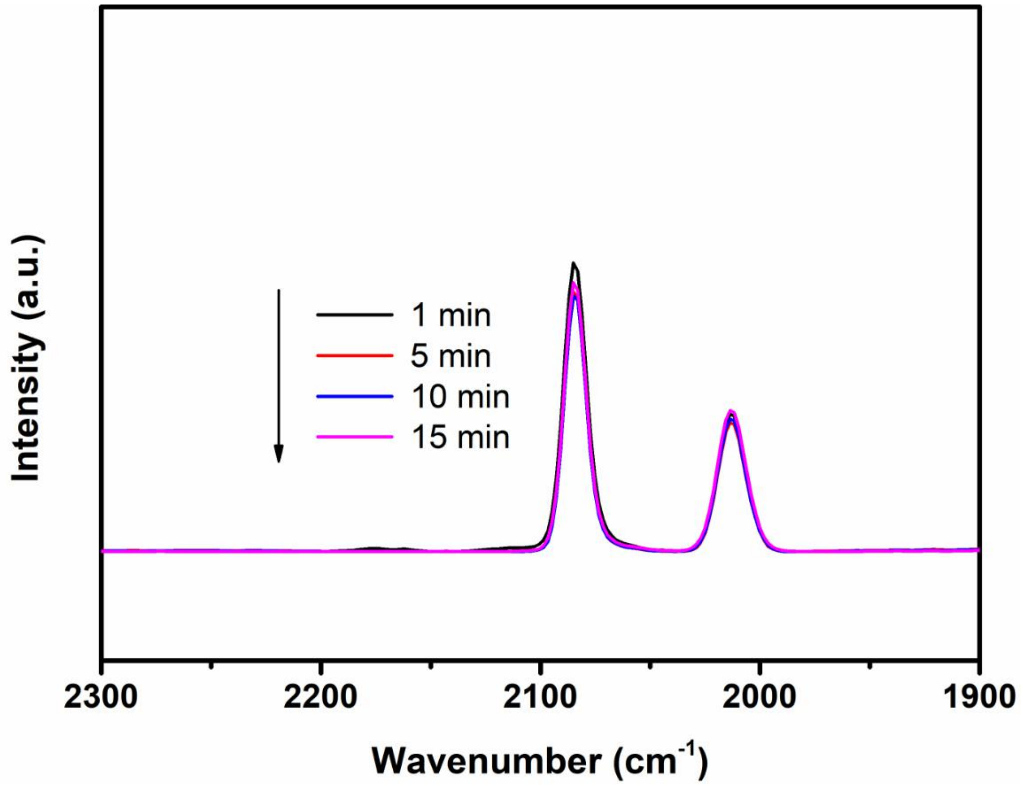

Supplementary Fig. 7. In situ CO-DRIFTs spectra of $[Rh(COD)Cl]_2$/ND under He with the different purging time.

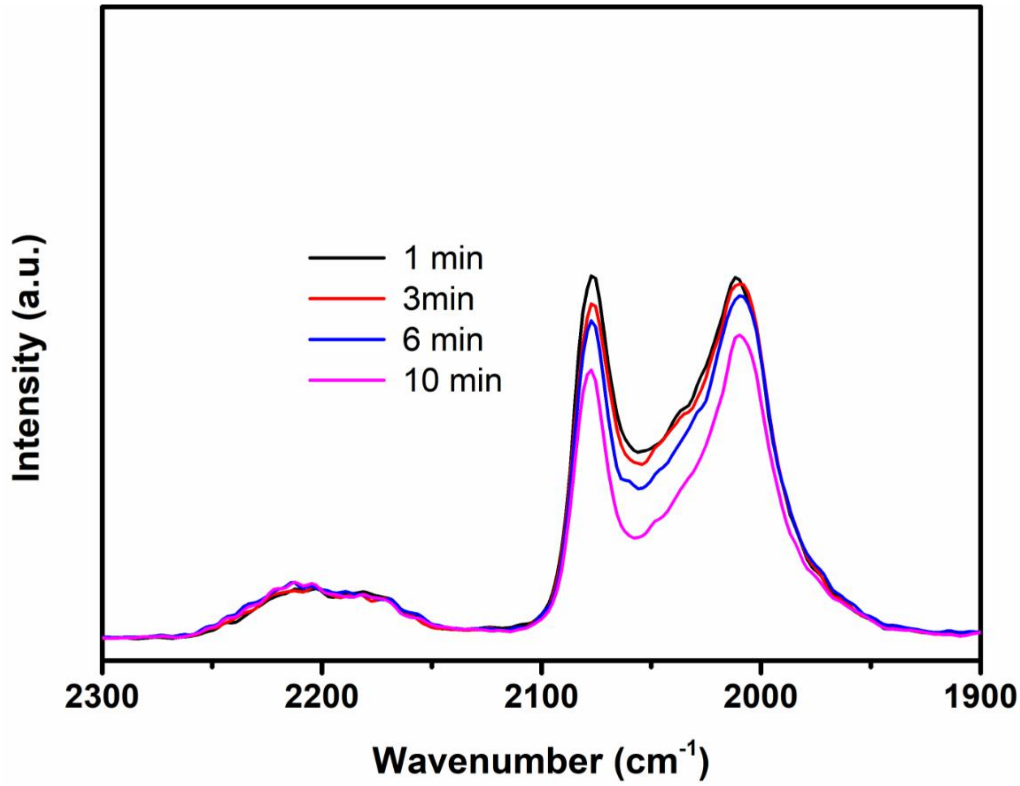

Supplementary Fig. 8. In situ CO-DRIFTs spectra of Rh NPs/ND under He with the different purging time.

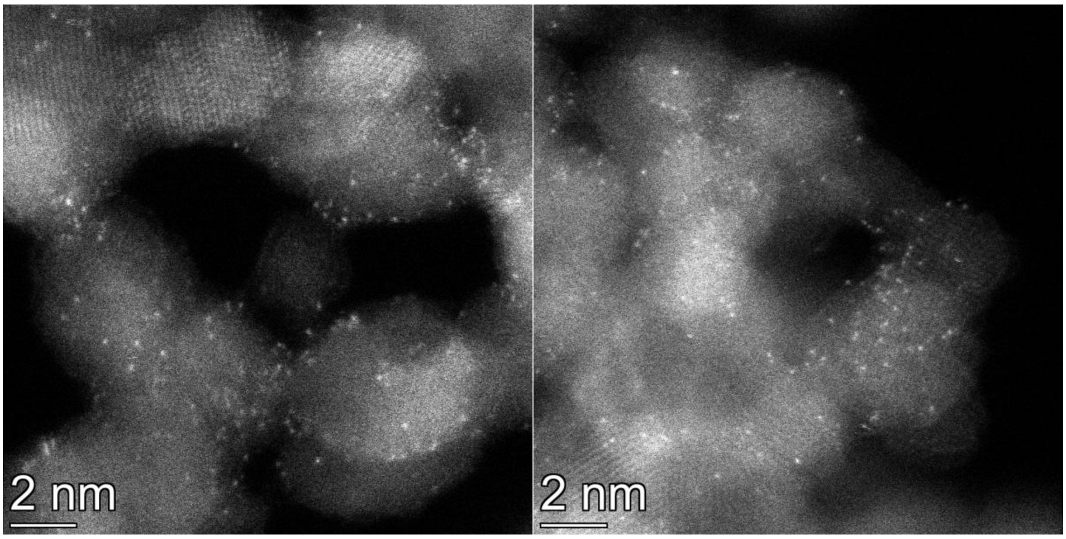

Supplementary Fig. 9. AC-HAADF-STEM images of the used Rh_1/PNP-ND after six runs.

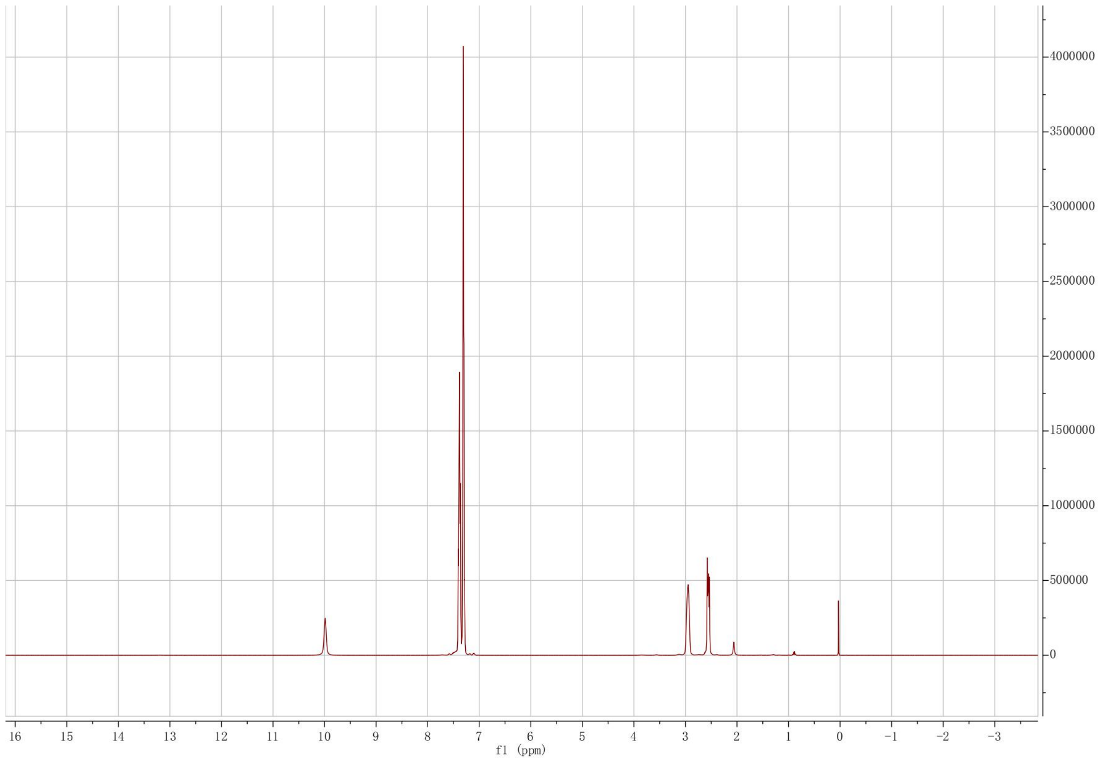

Supplementary Fig. 10. ^{1}H NMR of Bis[2-(diphenylphosphino)ethy]amine ·HCl

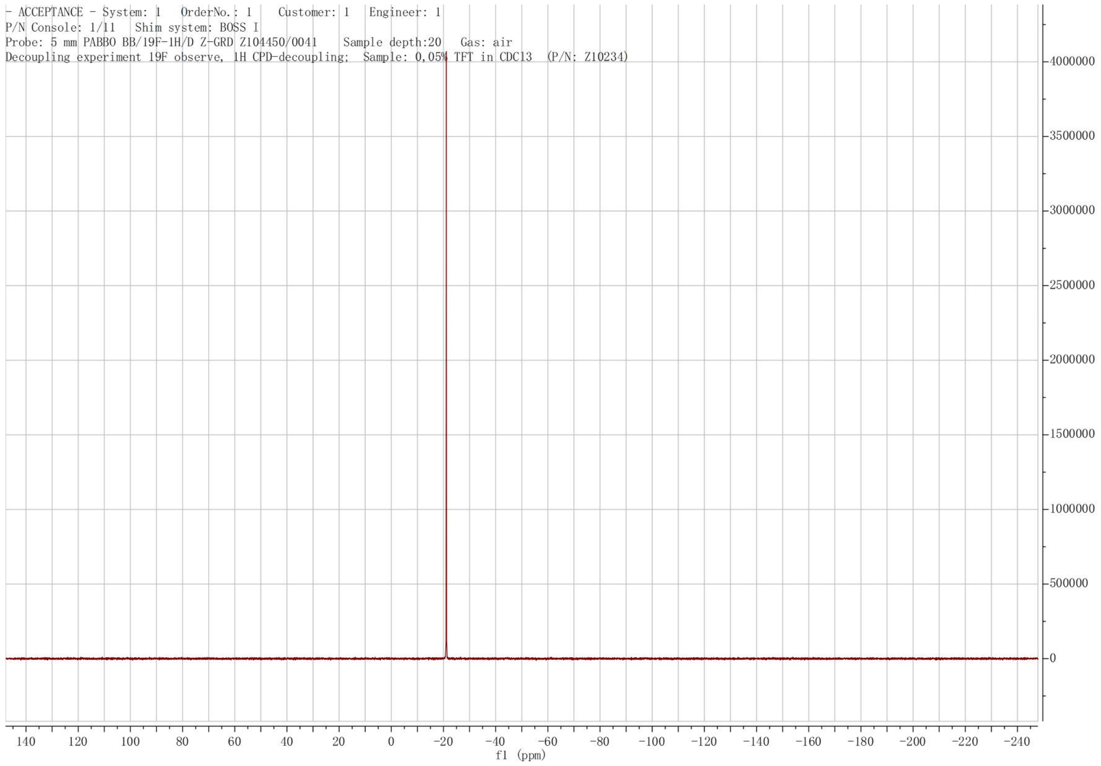

Supplementary Fig. 11. ^{31}P NMR of Bis[2-(diphenylphosphino)ethy]amine ·HCl

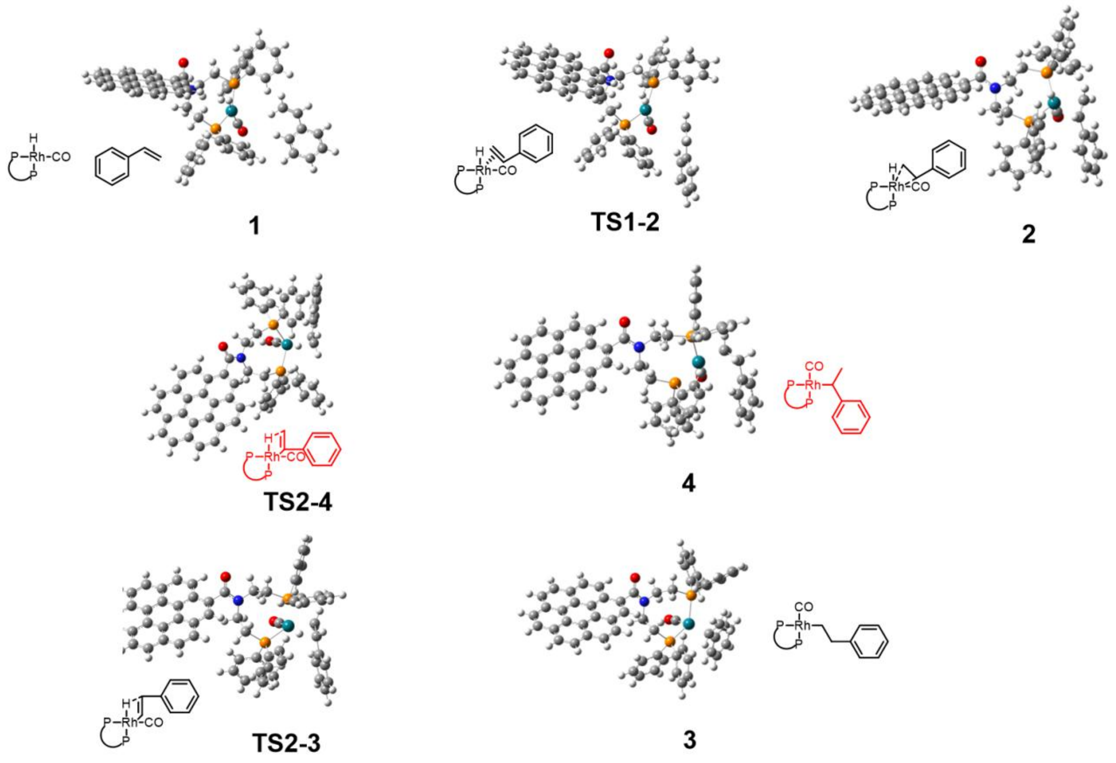

Supplementary Fig. 12. Structures of reaction intermediates and transition states in styrene hydroformylation. C (gray), H (white), O (red), N (blue), P (orange), and Rh (dark cyan).

Supplementary Note 1. Synthesis of Rh samples from different Rh precursors

50 mg PNP-ND was dispersed in 10 mL THF, and then 0.186 mmol Rh precursor was added. The slurry was stirred at 30 °C for 12 h under N_2. After that, the mixture was centrifugated, and washed with THF for three times. The solid was dried under vacuum at 50 °C overnight.

Supplementary Note 2. Synthesis of $[Rh(COD)Cl]_2$-PNP

THF solution (10 mL) containing $[Rh(COD)Cl]_2$ (23 mg, 0.0925 mmol), the diphosphino-amine (88 mg, 0.185 mmol) was added. The solution was heated at 30 °C for 12 h, and then the solvent was removed by rotary evaporation. The solid was dried under vacuum, denoted as $[Rh(COD)Cl]_2$-PNP.

Supplementary Note 3. Synthesis of Rh-PNP/ND

To a THF solution (10 mL) containing $[Rh(COD)Cl]_2$ (23 mg, 0.0925 mmol), the diphosphino-amine (88 mg, 0.185 mmol) was added. The solution was heated at 30 °C for 12 h, and then 50 mg ND was added. The slurry was stirred at 30 °C for 12 h. After centrifugation, gray solid was washed with THF. The solid was dried under vacuum at 50 °C overnight, and denoted as Rh-PNP/ND.

Supplementary Note 4. Synthesis of $[Rh(COD)Cl]_2$/ND

To a THF solution (10 mL) containing $[Rh(COD)Cl]_2$ (23 mg, 0.0925 mmol), 50 mg ND was added. The slurry was stirred at 30 °C for 12 h. After centrifugation, gray solid was washed with THF. The solid was dried under vacuum at 50 °C overnight, and denoted as $[Rh(COD)Cl]_2$/ND.

Supplementary Note 5. Synthesis of Rh NPs/ND

To a 10 mL THF solution containing $RhCl_3$ (3.86 mg, 0.0185 mmol), 50 mg ND was added. The slurry was stirred at 30 °C for 12 h. After centrifugation, gray solid was washed with THF for three times. The solid was dried under vacuum at 50 °C overnight, and denoted as $RhCl_3$/ND.

The synthesized $RhCl_3$/ND (50 mg) was hydrogenated under hydrogen at 200 °C with a flow rate of 20 mL min^{-1} for 1 h, and then deactivated under N_2 for 4 h. The above treatment gave the sample denoted as Rh NPs/ND.

Supplementary Note 6. Catalyst recycling experiment

After reaction, the reaction mixture containing Rh_1/PNP-ND was centrifugated, and the catalyst washed with ultra-pure water for three times. And then the gray solid was dispersed in 15 mL water with under ultrasonic treatment for 15 min. The slurry was loaded into the autoclave, and 3 mmol substrate and 15 mL toluene was added. After that the reactor was purged with syngas (CO/H_2=1), and charged with high-pressure syngas. The reactor was heated to 50 °C with stirring for 10 h.

Supplementary Note 7. Computational Details

All of the calculations were performed with Gaussian09 package[2]. Geometry optimization of all the minima was carried out at the B3LYP[3,4] level with the 6-31G* basis set for C, H, N, O, P and Lanl2DZ for Rh. Default convergence criteria were used. The vibrational frequency calculations were conducted at the same level of theory as geometry optimization to confirm whether each optimized structure is an

energy minimum. Single-point energies were evaluated with the larger 6-311+G(d,p) basis for all structures at their 6-31G* optimized geometries.

Supplementary Note 8. Coordinates in Fig. 6

1:

C	4.84210700	-2.99285200	-2.54975600
C	6.16238900	-2.71026200	-2.09892300
C	6.39904000	-1.50699200	-1.37861000
C	3.80600400	-2.13016500	-2.29712300
C	4.01426100	-0.92490700	-1.56975100
C	7.71538800	-1.19324800	-0.92561600
C	7.95035100	0.01779500	-0.20686900
C	5.32116300	-0.61008500	-1.11336600
C	5.55159100	0.60363900	-0.39587200
C	2.95469500	-0.02290400	-1.27696800
C	3.16233900	1.14748500	-0.58069200
C	9.26267500	0.33101400	0.24267100
C	9.47169800	1.54696100	0.95511100
C	6.87116900	0.91650800	0.05718000
C	7.11227800	2.12437100	0.76443400
C	4.48000200	1.50392200	-0.14012800
C	4.75609300	2.71250500	0.56189100
C	7.25568700	-3.58718500	-2.34686200
C	8.52220600	-3.28648800	-1.91194100
C	8.78969300	-2.08533900	-1.19321000
C	10.09606600	-1.74133200	-0.73358700
C	10.32228600	-0.57905400	-0.03868900
C	8.43641800	2.41231400	1.20346100

C	6.02274900	3.00777200	0.99834700
H	9.34790600	-3.96187800	-2.12269400
H	10.91511800	-2.42859900	-0.93023800
H	8.61412400	3.33818600	1.74354600
H	6.21172600	3.93771700	1.52860200
H	1.95855300	-0.26298900	-1.63910600
H	2.80318700	-2.36392900	-2.64605800
H	4.66584600	-3.91245900	-3.10204300
H	7.07503300	-4.50119800	-2.90673400
H	11.32407700	-0.33844900	0.30973800
H	10.47691200	1.78274300	1.29777700
H	3.95048700	3.42005800	0.72513000
C	2.01920000	2.11874500	-0.40487200
O	2.02838700	3.19796800	-0.99589500
N	0.95825200	1.74572700	0.39054100
C	1.10242600	0.72392200	1.44341700
H	2.17118100	0.60973700	1.63938400
H	0.66912300	1.12361600	2.36689500
C	-0.11305600	2.75507900	0.49146000
H	-0.76749100	2.42627600	1.29988200
H	0.34161400	3.71165500	0.77376500
C	-0.91964200	2.94868800	-0.81996500
H	-0.49238400	2.32159600	-1.60617000
H	-0.85068900	3.98819400	-1.15356600
C	0.54157500	-0.69319400	1.15970700
H	0.63338500	-0.91802900	0.09479400
H	1.16923000	-1.40500700	1.71002800
P	-2.71296300	2.42457300	-0.67381700
P	-1.23931800	-1.02916600	1.62910400
C	-1.47492600	-2.79130000	1.13205600

C	-0.43415000	-3.65636600	0.76147800
C	-2.79242700	-3.28026000	1.11461500
C	-0.70596700	-4.97407100	0.38196800
H	0.59569300	-3.31361400	0.75186100
C	-3.06297800	-4.59703700	0.74565700
H	-3.60887500	-2.61502200	1.38461700
C	-2.01775200	-5.44824700	0.37534900
H	0.11177700	-5.62764000	0.08923000
H	-4.08951300	-4.95327000	0.73422100
H	-2.22539000	-6.47260200	0.07651100
C	-1.09239900	-1.12628800	3.47511200
C	-1.44195800	-0.00779800	4.24675800
C	-0.58851500	-2.26331200	4.12827500
C	-1.27960300	-0.01879100	5.63479700
H	-1.85201900	0.87142000	3.75619300
C	-0.43495700	-2.27805400	5.51502800
H	-0.32688700	-3.14727300	3.55305100
C	-0.77723000	-1.15523700	6.27246300
H	-1.55388800	0.85956400	6.21501900
H	-0.05200100	-3.16997100	6.00361900
H	-0.65912700	-1.16870900	7.35230600
C	-3.26479900	2.27770600	-2.42401600
C	-4.30927300	1.38504000	-2.71195900
C	-2.68189900	3.00291800	-3.47773000
C	-4.77327100	1.22968800	-4.01878600
H	-4.75070900	0.79891200	-1.91112200
C	-3.13689500	2.83815600	-4.78824500
H	-1.86651300	3.69476100	-3.28300400
C	-4.18638800	1.95495100	-5.05963200
H	-5.57677300	0.52703700	-4.22132500

H	-2.67468400	3.40064800	-5.59483200
H	-4.54123300	1.82823800	-6.07934800
C	-3.53999400	3.97164500	-0.06953500
C	-4.10526900	4.92410900	-0.93167100
C	-3.56606700	4.21872900	1.31221000
C	-4.67379900	6.09400100	-0.42351600
H	-4.11105500	4.75282500	-2.00336100
C	-4.12431400	5.39266800	1.81879300
H	-3.15828900	3.47960500	1.99554200
C	-4.68112400	6.33423900	0.95136200
H	-5.11313900	6.81725300	-1.10599700
H	-4.13414800	5.56516600	2.89176000
H	-5.12476300	7.24508900	1.34467800
Rh	-2.64742200	0.52745000	0.66675700
H	-1.64683300	0.14253700	-0.58319800
C	-3.99214700	0.83080700	1.99409800
O	-4.88094600	0.93449500	2.72979900
C	-8.16754300	-3.71017500	-2.19055100
C	-9.00756000	-4.10952600	-1.15043600
C	-8.57541900	-4.01590700	0.17231500
C	-7.29901600	-3.51160200	0.44451200
C	-6.46202100	-3.11289600	-0.59445700
C	-6.87834800	-3.21208100	-1.93595000
H	-8.51120300	-3.78649400	-3.22092300
H	-9.99965600	-4.49206600	-1.37504900
H	-9.22737200	-4.32220200	0.98616600
H	-6.95989300	-3.41923100	1.47467100
H	-5.48111500	-2.70718000	-0.36400900
C	-6.02699000	-2.81805900	-3.07175200
H	-6.53050100	-2.82879400	-4.03968900

C	-4.73167600	-2.47597800	-3.03711500
H	-4.14734800	-2.46160800	-2.12158300
H	-4.20363500	-2.20107600	-3.94556200

TS 1-2：

C	5.23173300	-2.32373700	-3.04952100
C	6.47574900	-2.14962800	-2.37837100
C	6.58301000	-1.13773000	-1.38499200
C	4.14653600	-1.53879400	-2.75000600
C	4.22568700	-0.52230400	-1.75540200
C	7.81883700	-0.93858200	-0.69985600
C	7.92580500	0.08176400	0.29240000
C	5.45490400	-0.32016500	-1.07386600
C	5.55790500	0.70287100	-0.08218200
C	3.11177000	0.29186800	-1.41207900
C	3.19522200	1.27489100	-0.45191400
C	9.15806900	0.28103000	0.97351200
C	9.24114400	1.30849000	1.95631700
C	6.79851000	0.90371400	0.59861500
C	6.91350300	1.92341000	1.58183100
C	4.43629200	1.52376500	0.22435400
C	4.58667200	2.54716000	1.20538000
C	7.61717800	-2.95122700	-2.66655600
C	8.80493500	-2.76136200	-2.00544100
C	8.94189600	-1.75437900	-1.00749600
C	10.16216900	-1.53327100	-0.30756100
C	10.26580900	-0.55246700	0.64701500
C	8.15966300	2.10070200	2.24855900
C	5.77887500	2.73572000	1.85840300
H	9.66567100	-3.38330500	-2.23925900
H	11.01957300	-2.15874800	-0.54407600
H	8.24125100	2.88284900	2.99941900
H	5.87163600	3.52549900	2.59996000

H	2.17550600	0.13563300	-1.94199300
H	3.20452500	-1.68425800	-3.27332600
H	5.15416300	-3.09577500	-3.81127200
H	7.53244100	-3.72389600	-3.42689700
H	11.20601700	-0.39705700	1.17065900
H	10.18498200	1.45830700	2.47503000
H	3.74723000	3.20195700	1.41255600
C	2.00720900	2.17160900	-0.19048300
O	2.02579600	3.34612800	-0.55719900
N	0.90790100	1.63655800	0.44040300
C	0.97021600	0.37841800	1.20116700
H	2.01838900	0.20221700	1.45348200
H	0.45251400	0.53608300	2.15310400
C	-0.18983300	2.58968700	0.68412100
H	-0.87017100	2.10510700	1.38687400
H	0.22811100	3.48317400	1.16010000
C	-0.94607800	3.01405200	-0.60273600
H	-0.52492300	2.47396700	-1.45279700
H	-0.81977400	4.08457200	-0.78937000
C	0.44442700	-0.90343200	0.50335900
H	0.57809200	-0.81370200	-0.57687400
H	1.06129700	-1.74143000	0.85018100
P	-2.75661700	2.55940400	-0.53136000
P	-1.34677900	-1.37295700	0.79359200
C	-1.49576000	-2.98300400	-0.10145400
C	-0.42559000	-3.64762600	-0.72011500
C	-2.77430400	-3.56272300	-0.16514300
C	-0.62911500	-4.86405500	-1.37809100
H	0.57456200	-3.22764000	-0.69948500
C	-2.97392200	-4.78253900	-0.80989800

H	-3.62101900	-3.05063800	0.28287300
C	-1.90036900	-5.43628200	-1.42033900
H	0.21082500	-5.36265600	-1.85507300
H	-3.97159600	-5.21106600	-0.84386000
H	-2.05566300	-6.38330500	-1.93054300
C	-1.26253900	-1.89953400	2.57151200
C	-1.65554700	-0.99960000	3.57328700
C	-0.75854100	-3.15378500	2.95322300
C	-1.54178400	-1.33929000	4.92334100
H	-2.06201700	-0.03294500	3.29006600
C	-0.65250900	-3.49603600	4.30167100
H	-0.45798600	-3.87109200	2.19508300
C	-1.04173000	-2.58911600	5.29034900
H	-1.85277500	-0.62974300	5.68562100
H	-0.26827200	-4.47397600	4.57999700
H	-0.96040900	-2.85838600	6.34009700
C	-3.40914600	3.00853900	-2.19969500
C	-4.80157900	2.97224700	-2.39190300
C	-2.59039800	3.33466500	-3.29040000
C	-5.35872800	3.25897300	-3.63678400
H	-5.45412100	2.72250400	-1.55880100
C	-3.14994300	3.61675300	-4.54013400
H	-1.51207200	3.37505100	-3.17797300
C	-4.53258300	3.58051600	-4.71737900
H	-6.43758300	3.22825500	-3.76413500
H	-2.49906300	3.86836300	-5.37341300
H	-4.96534300	3.80023600	-5.68961300
C	-3.43839800	3.92983100	0.51957800
C	-3.53146600	5.24254800	0.02494600
C	-3.81206000	3.68513200	1.84732700

C	-3.98813400	6.27845700	0.83873900
H	-3.25466400	5.45457400	-1.00403300
C	-4.26755100	4.72346400	2.66444300
H	-3.75165500	2.67627000	2.24181500
C	-4.35748400	6.02129000	2.16196200
H	-4.05768200	7.28690200	0.43921100
H	-4.55656200	4.51324600	3.69085000
H	-4.71597000	6.82894500	2.79469800
Rh	-2.77638500	0.34413200	0.19983500
H	-1.74874400	0.29588100	-1.08319400
C	-4.14744900	0.23813500	1.52718500
O	-5.06340100	0.08972800	2.22424400
C	-6.77488100	-4.21900200	-1.51950200
C	-7.48760900	-5.19954700	-0.82901800
C	-7.97171700	-4.93868700	0.45414700
C	-7.73213600	-3.69126300	1.03878500
C	-7.02227400	-2.71161700	0.34828100
C	-6.53692100	-2.95407400	-0.95111100
H	-6.40454000	-4.42558500	-2.52137300
H	-7.66508200	-6.16616900	-1.29365000
H	-8.52369200	-5.70101200	0.99733200
H	-8.09290600	-3.48344400	2.04283000
H	-6.82307300	-1.75816100	0.82859700
C	-5.79200300	-1.94723800	-1.72620800
H	-5.24686200	-2.33999300	-2.58433100
C	-5.74230100	-0.62804600	-1.49374900
H	-6.28597700	-0.15983400	-0.67783100
H	-5.16083500	0.03145300	-2.12907200

2：

C	-5.66132500	-2.00621000	2.93654500
C	-6.86553600	-1.77753800	2.21130800
C	-6.85308600	-0.84686100	1.13600400
C	-4.50147200	-1.34897700	2.61192200
C	-4.46122600	-0.41564000	1.53670400
C	-8.04652400	-0.59757700	0.39474200
C	-8.03192500	0.33939100	-0.68152400
C	-5.64696200	-0.16172000	0.79819900
C	-5.62666900	0.77834600	-0.27765700
C	-3.26987600	0.26567500	1.16910100
C	-3.23223500	1.17077700	0.13086700
C	-9.22221000	0.58862700	-1.41883500
C	-9.18465800	1.53024900	-2.48654100
C	-6.82571000	1.02763900	-1.01598100
C	-6.82104700	1.96309000	-2.08531300
C	-4.42649000	1.46841000	-0.61158100
C	-4.45716200	2.40785700	-1.68390800
C	-8.08270000	-2.44736600	2.52447800
C	-9.23007600	-2.20839100	1.81015700
C	-9.24752900	-1.28099400	0.72943500
C	-10.42334000	-1.01210900	-0.02747100
C	-10.41047500	-0.11099300	-1.06257700
C	-8.02714400	2.19324200	-2.80711600
C	-5.60918100	2.64115500	-2.39165500
H	-10.15011400	-2.72930100	2.06422500
H	-11.34064000	-1.53593100	0.23031700
H	-8.01537800	2.91121000	-3.62355100
H	-5.60808400	3.36518800	-3.20292700

H	-2.36732200	0.06599200	1.74116800
H	-3.59038800	-1.53532700	3.17538500
H	-5.67531300	-2.71702900	3.75918800
H	-8.08915800	-3.15824500	3.34721800
H	-11.31793200	0.08288000	-1.62941000
H	-10.09673000	1.71843200	-3.04781800
H	-3.55662800	2.96276400	-1.91775400
C	-1.96571300	1.94256300	-0.15045700
O	-1.96118900	3.17286000	-0.08586700
N	-0.81769200	1.25030700	-0.45767300
C	-0.74447600	-0.17495400	-0.77690100
H	-1.76466000	-0.53748200	-0.90773900
H	-0.25210900	-0.27542600	-1.75120700
C	0.37765600	2.06356700	-0.70614000
H	1.06824700	1.43665200	-1.27669200
H	0.08873600	2.91390500	-1.32912500
C	1.06138600	2.56880300	0.58202000
H	0.72187800	1.97423100	1.43619300
H	0.78980000	3.60643100	0.79816200
C	-0.03584500	-1.06012600	0.28660600
H	-0.00839000	-0.53518900	1.24630600
H	-0.62357100	-1.97016100	0.45280200
P	2.90856100	2.31105400	0.55289000
P	1.74716300	-1.58022100	-0.04817300
C	1.84832000	-2.94884300	1.21055500
C	1.89466400	-2.61744700	2.57569600
C	1.87374200	-4.30575200	0.85436600
C	1.94837900	-3.61050500	3.55338500
H	1.90661900	-1.57367700	2.87521700
C	1.94186500	-5.30028100	1.83454100

H	1.84257700	-4.59485000	-0.19040700
C	1.97550900	-4.95823100	3.18609700
H	1.97898900	-3.32999800	4.60320600
H	1.96639200	-6.34498800	1.53503100
H	2.02691300	-5.73252400	3.94689000
C	1.59485300	-2.52338300	-1.63676000
C	2.76226000	-2.77923900	-2.37387400
C	0.37578600	-3.02689700	-2.12334500
C	2.71305300	-3.50849200	-3.56311400
H	3.71820200	-2.41874300	-2.00996300
C	0.32413300	-3.74370700	-3.32031700
H	-0.54548600	-2.87402300	-1.57023200
C	1.49301700	-3.98537700	-4.04446800
H	3.63225400	-3.69804500	-4.11017900
H	-0.63061300	-4.11563500	-3.68296300
H	1.45210100	-4.54371000	-4.97596100
C	3.38978400	3.09577300	2.16686800
C	3.52893000	4.48842200	2.29282800
C	3.57801800	2.29914700	3.30651100
C	3.84140400	5.06475200	3.52459900
H	3.39720000	5.12845900	1.42604600
C	3.88558700	2.87693900	4.54003100
H	3.48473200	1.22112900	3.22044100
C	4.02004400	4.26119400	4.65251900
H	3.94636800	6.14390600	3.60125200
H	4.02804700	2.24176400	5.41057800
H	4.26627800	4.71124800	5.61064300
C	3.51356000	3.55784100	-0.67356700
C	2.69814000	4.54962100	-1.24071200
C	4.86623300	3.50797100	-1.05188800

C	3.21994900	5.46162400	-2.16269000
H	1.65084400	4.62583500	-0.96675800
C	5.38975600	4.42513300	-1.96146500
H	5.50926600	2.73996500	-0.63094100
C	4.56580200	5.40364600	-2.52360600
H	2.57025600	6.21810400	-2.59500600
H	6.43929400	4.37062900	-2.23823900
H	4.97045500	6.11320500	-3.24028900
Rh	3.51422700	0.03405000	0.16950300
H	3.05829400	-0.09007500	1.71502900
C	3.98702200	0.26287200	-1.69828300
O	4.24148700	0.43977200	-2.81083100
C	5.62618000	-3.63608800	-0.01713700
C	6.13562900	-4.55623700	-0.93276900
C	6.87655900	-4.11989200	-2.03293500
C	7.10406900	-2.75007000	-2.19914900
C	6.59648100	-1.83128900	-1.28315400
C	5.83864400	-2.25234000	-0.17249000
H	5.04760600	-3.98783500	0.83356000
H	5.95385900	-5.61788600	-0.78408200
H	7.27683400	-4.83458400	-2.74702900
H	7.68506700	-2.39422700	-3.04655100
H	6.78829500	-0.77337500	-1.43623600
C	5.29581200	-1.32574100	0.84401200
H	4.88149900	-1.82615100	1.71544400
C	5.62045100	0.03161500	0.97847900
H	6.32217500	0.50063600	0.29385700
H	5.55632700	0.50387300	1.95260600

TS 2-4:

C	-5.94011500	-2.19110200	2.58608200
C	-7.10661700	-1.68491200	1.94469400
C	-6.98578700	-0.54813500	1.09862600
C	-4.71436100	-1.60417700	2.39667000
C	-4.56535600	-0.46818500	1.55036300
C	-8.13901500	-0.01944500	0.44553400
C	-8.01547400	1.12359700	-0.40002600
C	-5.71100700	0.06385300	0.90214100
C	-5.58162200	1.20856200	0.05696000
C	-3.30455800	0.14570700	1.32031600
C	-3.16206200	1.24452900	0.50186700
C	-9.16550000	1.65084600	-1.04953500
C	-9.01845400	2.79401300	-1.88601300
C	-6.74039500	1.73815100	-0.59211600
C	-6.62644100	2.87908100	-1.43124400
C	-4.31184500	1.82325200	-0.13670200
C	-4.23250200	2.97322300	-0.97604300
C	-8.39042200	-2.27605400	2.11980900
C	-9.49910900	-1.76835300	1.48980200
C	-9.40829500	-0.63042800	0.63815700
C	-10.54163400	-0.08151500	-0.02663200
C	-10.42396900	1.01791600	-0.83973500
C	-7.79456600	3.38638400	-2.06906600
C	-5.34680600	3.47674800	-1.59846400
H	-10.47115000	-2.23334000	1.63582200
H	-11.51126100	-0.55060900	0.12197900
H	-7.69960300	4.26017300	-2.70911800
H	-5.26148700	4.35709200	-2.23096500

H	-2.43266400	-0.27056500	1.81825600
H	-3.83352500	-2.00395600	2.89335000
H	-6.03646200	-3.05898700	3.23397700
H	-8.47983500	-3.14495600	2.76738100
H	-11.30025600	1.42444300	-1.33895500
H	-9.89989500	3.19470200	-2.38084200
H	-3.27583100	3.46776000	-1.09561100
C	-1.81479000	1.91318800	0.36035700
O	-1.66063200	3.07829700	0.72825700
N	-0.77347800	1.19307700	-0.17604800
C	-0.89422400	-0.12730600	-0.79773000
H	-1.95046300	-0.30562300	-1.00247500
H	-0.39276400	-0.08209000	-1.77156100
C	0.50988400	1.89526600	-0.30482000
H	1.05062400	1.40230700	-1.11623300
H	0.30192900	2.92445100	-0.60525900
C	1.36369200	1.89872500	0.98335600
H	1.00909100	1.12163300	1.66742400
H	1.26301300	2.84888600	1.51681000
C	-0.33945700	-1.30818800	0.04808000
H	-0.16408900	-0.97114200	1.07356800
H	-1.08863200	-2.10550500	0.11560300
P	3.15086200	1.45359000	0.63710100
P	1.29801400	-2.04796000	-0.52351000
C	1.31611300	-3.53877500	0.58853700
C	1.38410200	-3.34798700	1.98039300
C	1.30484100	-4.85402900	0.09940300
C	1.42150600	-4.43408900	2.85405700
H	1.41550000	-2.34032300	2.38702700
C	1.35414800	-5.94253100	0.97473500

H	1.25248000	-5.03472700	-0.96917600
C	1.40823600	-5.73853700	2.35343900
H	1.46654300	-4.26139800	3.92642100
H	1.34549300	-6.95255300	0.57297300
H	1.44349900	-6.58599800	3.03266900
C	0.83348300	-2.78876600	-2.16151900
C	1.86043200	-3.08769100	-3.07125400
C	-0.48855000	-3.09118000	-2.53003800
C	1.57918600	-3.67245800	-4.30710600
H	2.88662300	-2.85282100	-2.80375500
C	-0.77319300	-3.66300500	-3.77133100
H	-1.31107700	-2.88885000	-1.85155100
C	0.25996300	-3.95682300	-4.66311800
H	2.39093600	-3.89457100	-4.99465600
H	-1.80390900	-3.88061300	-4.03894300
H	0.03734300	-4.40144600	-5.62939500
C	3.88783200	1.71258600	2.32203000
C	4.79340600	2.74415300	2.61377900
C	3.55084500	0.81236000	3.34807500
C	5.33843300	2.87406200	3.89400000
H	5.07587800	3.45105600	1.84083700
C	4.08456400	0.94978400	4.62909300
H	2.86814200	-0.00809800	3.13986700
C	4.98461700	1.98205700	4.90653300
H	6.03888000	3.68000800	4.09806800
H	3.80285000	0.24697800	5.40919300
H	5.40769400	2.08638800	5.90202600
C	3.76776900	2.91774000	-0.31254700
C	3.18190200	4.19214000	-0.22544500
C	4.87752200	2.74547600	-1.15533700

C	3.68735200	5.26104200	-0.96756200
H	2.32828200	4.36235500	0.42366100
C	5.39082100	3.81786200	-1.88703500
H	5.34063400	1.76693800	-1.23669300
C	4.79405900	5.07660200	-1.79907400
H	3.21562200	6.23753300	-0.89404600
H	6.25283000	3.66357100	-2.53027200
H	5.18640600	5.90940200	-2.37689900
Rh	3.28105300	-0.72277700	-0.41207400
H	3.40486000	-1.29339500	1.15571400
C	3.33544200	-0.17604600	-2.22731300
O	3.34845500	0.18006700	-3.33141100
C	7.30905400	-0.67088900	-1.55947500
C	8.40679400	0.18056300	-1.67571600
C	8.77835100	0.99962700	-0.60714900
C	8.03455900	0.95463600	0.57587300
C	6.93624100	0.10585400	0.69123800
C	6.54817000	-0.72775400	-0.37597800
H	7.02942100	-1.30556700	-2.39784800
H	8.97473200	0.20094500	-2.60258100
H	9.63430800	1.66332600	-0.69389900
H	8.30772100	1.58856100	1.41564700
H	6.36539800	0.10524900	1.61461000
C	5.41685400	-1.67260800	-0.29909000
H	5.34478800	-2.36169800	-1.13671500
C	4.75979000	-2.05774700	0.89021500
H	5.13736200	-1.68749200	1.84144100
H	4.32225000	-3.05060500	0.94013500

TS 2-3:

C	-5.45218900	-2.26153100	2.83462800
C	-6.66229700	-2.00101100	2.13029000
C	-6.69135000	-0.93747300	1.18666400
C	-4.32605900	-1.50893900	2.61443500
C	-4.32787300	-0.44002900	1.67300700
C	-7.89126300	-0.65367800	0.46840300
C	-7.91879000	0.41670300	-0.47516800
C	-5.52084100	-0.15261000	0.95870700
C	-5.54439400	0.92238900	0.01800600
C	-3.17270100	0.34792700	1.41764500
C	-3.18017900	1.38624300	0.51269800
C	-9.11515100	0.69956700	-1.19004100
C	-9.11932700	1.77491800	-2.12377800
C	-6.74886600	1.20519600	-0.69838800
C	-6.78567900	2.27445900	-1.63338200
C	-4.38209300	1.71445600	-0.20148600
C	-4.45412400	2.78749900	-1.13731500
C	-7.84518300	-2.76670900	2.33725400
C	-8.99873800	-2.49439800	1.64515300
C	-9.05713200	-1.43537200	0.69461600
C	-10.24012100	-1.12961200	-0.03671400
C	-10.26735500	-0.10096800	-0.94495700
C	-7.99668300	2.53438700	-2.33670600
C	-5.61053200	3.05220100	-1.82677100
H	-9.89227400	-3.08980300	1.81674700
H	-11.13059100	-1.72874400	0.13775600
H	-8.01762800	3.35402900	-3.05081500
H	-5.64268800	3.87786100	-2.53350600

H	-2.26544000	0.12388600	1.97266500
H	-3.41029600	-1.72125400	3.16096900
H	-5.43398600	-3.07421200	3.55680000
H	-7.81999900	-3.57865200	3.06004900
H	-11.17988400	0.11903900	-1.49383600
H	-10.03569200	1.98818100	-2.66888400
H	-3.58294600	3.41640000	-1.28005300
C	-1.96301800	2.27081300	0.37254000
O	-2.02380300	3.45086600	0.71508800
N	-0.80458700	1.71902100	-0.12602600
C	-0.70144100	0.37474400	-0.69892100
H	-1.70427600	0.05199400	-0.98305900
H	-0.12424500	0.46283800	-1.62577600
C	0.36662400	2.59834700	-0.21817400
H	0.90556900	2.31957900	-1.12848500
H	-0.00371100	3.61687400	-0.33704400
C	1.30119900	2.53039500	1.01843400
H	0.94950600	1.74625500	1.69347700
H	1.26888800	3.47038300	1.57928400
C	-0.06457500	-0.69026000	0.23767700
H	0.13710200	-0.25058700	1.21805600
H	-0.76695300	-1.51129300	0.42242900
P	3.06543400	2.11229000	0.54161900
P	1.58274300	-1.40897200	-0.31002700
C	1.83585400	-2.57479100	1.11511200
C	2.54422800	-2.13590700	2.24454000
C	1.28140800	-3.86433300	1.12967700
C	2.68904000	-2.96068200	3.36106600
H	2.99418100	-1.14718000	2.24637400
C	1.43658400	-4.69316200	2.24276600

H	0.73271800	-4.22930700	0.26709600
C	2.13870500	-4.24389900	3.36216500
H	3.24102400	-2.60138500	4.22582900
H	1.00888200	-5.69242300	2.23156700
H	2.26003400	-4.89054000	4.22727000
C	1.09827000	-2.55118100	-1.68382200
C	2.13169900	-3.21401800	-2.36850500
C	-0.22616100	-2.78563700	-2.08290100
C	1.84652100	-4.09460600	-3.41025200
H	3.16419500	-3.03326200	-2.08311500
C	-0.51043700	-3.65462100	-3.14079100
H	-1.05307300	-2.29698800	-1.57807100
C	0.52358200	-4.31358000	-3.80436400
H	2.65992900	-4.60314400	-3.92104500
H	-1.54265900	-3.81396700	-3.44143000
H	0.30188300	-4.99013900	-4.62541300
C	3.94329700	2.16758300	2.17003200
C	5.32178700	2.45302400	2.20017200
C	3.32246600	1.79051200	3.37307100
C	6.04832600	2.36838600	3.38744700
H	5.82893600	2.76173300	1.28983600
C	4.05065500	1.70668000	4.56249900
H	2.26084800	1.56444500	3.39655800
C	5.41589300	1.99339600	4.57565900
H	7.11018000	2.60115400	3.38472200
H	3.54390000	1.42287900	5.48145900
H	5.98117800	1.92965500	5.50140400
C	3.58153200	3.72933600	-0.21565600
C	3.67449100	4.91024600	0.54220100
C	3.83564600	3.79417300	-1.59255000

C	4.01182100	6.11979300	-0.06341100
H	3.48975300	4.88266000	1.61268400
C	4.16940400	5.00758100	-2.20152500
H	3.76960600	2.88776300	-2.18625600
C	4.25980000	6.17121400	-1.43850700
H	4.07989100	7.02338700	0.53695700
H	4.36077300	5.03848900	-3.27096600
H	4.52240600	7.11484200	-1.90953400
Rh	3.35258700	0.08112100	-0.84035200
H	4.18222800	-0.54499400	0.45859000
C	2.77027500	0.36144000	-2.63739000
O	2.44303900	0.52576700	-3.73987000
C	6.38606500	-2.83961400	0.32401300
C	6.75797900	-4.15786900	0.05737900
C	6.69908600	-4.65306400	-1.24534400
C	6.25971700	-3.81503600	-2.27588600
C	5.87954500	-2.50250700	-2.00645000
C	5.93852600	-1.98818200	-0.69898200
H	6.44122500	-2.46114700	1.34248000
H	7.09749900	-4.79551900	0.86955000
H	6.99113000	-5.67777000	-1.45821300
H	6.21373500	-4.18675800	-3.29660200
H	5.52353000	-1.87270900	-2.81614800
C	5.60800500	-0.57480400	-0.36313500
H	6.00818900	-0.27123300	0.60428800
C	5.46038400	0.47728400	-1.31583500
H	5.61806900	0.25962200	-2.36774600
H	5.76539000	1.47779300	-1.02161500

4:

C	-5.90201700	-2.34345400	2.78750500
C	-7.08758500	-2.04440000	2.05717300
C	-7.04816900	-1.01457600	1.07717300
C	-4.73448300	-1.66040700	2.55692600
C	-4.66718400	-0.62770500	1.57825100
C	-8.22210400	-0.69371300	0.33220300
C	-8.18078400	0.34251900	-0.64817900
C	-5.83448100	-0.30173100	0.83836400
C	-5.78842900	0.73813100	-0.14031000
C	-3.46721800	0.08378000	1.30726500
C	-3.40629900	1.08541500	0.36342800
C	-9.35127500	0.66216200	-1.38986300
C	-9.28671200	1.70226700	-2.36051600
C	-6.96762700	1.05916100	-0.88237100
C	-6.93581800	2.09415400	-1.85532200
C	-4.58139500	1.45598900	-0.37450500
C	-4.58480200	2.49697000	-1.34881100
C	-8.31174200	-2.73951100	2.27284500
C	-9.44022500	-2.43176200	1.55473400
C	-9.43040400	-1.40500500	0.56779700
C	-10.58604800	-1.06401100	-0.19113000
C	-10.54721700	-0.06787500	-1.13447000
C	-8.12253000	2.39300100	-2.58404200
C	-5.71788900	2.79937500	-2.06099100
H	-10.36589700	-2.97363000	1.73317500
H	-11.50890400	-1.60959400	-0.00960600
H	-8.09082300	3.18631000	-3.32688400
H	-5.69713600	3.59966200	-2.79675200

H	-2.57725200	-0.17434300	1.87643700
H	-3.83813200	-1.90174900	3.12326000
H	-5.93633300	-3.12971600	3.53777000
H	-8.33881300	-3.52601500	3.02314600
H	-11.43953300	0.17990400	-1.70429900
H	-10.18353600	1.94403500	-2.92581900
H	-3.67929500	3.07208000	-1.50311800
C	-2.13434900	1.88186600	0.18913900
O	-2.12005400	3.08418900	0.45053400
N	-1.00283800	1.23400600	-0.25093600
C	-0.94273900	-0.15007800	-0.71822300
H	-1.96645500	-0.51228200	-0.81911400
H	-0.50318300	-0.14804900	-1.72409800
C	0.21700700	2.03465200	-0.37984100
H	0.81293100	1.57815700	-1.17662800
H	-0.06561000	3.04186800	-0.68945800
C	1.04491800	2.10794600	0.92469200
H	0.81827000	1.24223600	1.55255800
H	0.79183400	3.00701000	1.49541300
C	-0.15808600	-1.10721300	0.21816300
H	-0.18036700	-0.71324200	1.23822800
H	-0.66335400	-2.07892100	0.26210600
P	2.86714700	2.03192400	0.53859000
P	1.66076200	-1.43667900	-0.17901500
C	2.12068800	-2.28716700	1.40487500
C	2.97777600	-1.64062800	2.30743900
C	1.59895300	-3.54414700	1.75291800
C	3.30001700	-2.22952800	3.53303000
H	3.39984500	-0.67452700	2.04690700
C	1.92286800	-4.13383200	2.97510100

H	0.94776100	-4.07135400	1.06115300
C	2.77319900	-3.47713500	3.86890500
H	3.96421700	-1.71088500	4.21917000
H	1.51380300	-5.10855700	3.22824400
H	3.02635900	-3.93918000	4.81948100
C	1.53695100	-2.86802200	-1.34911700
C	2.70037900	-3.62009200	-1.59352800
C	0.36567200	-3.20576400	-2.04222600
C	2.68427800	-4.68513700	-2.49126300
H	3.62737600	-3.36821100	-1.08542000
C	0.35422100	-4.26764800	-2.95220900
H	-0.55244100	-2.64733600	-1.88950600
C	1.51076200	-5.01228000	-3.17638800
H	3.59500200	-5.25260800	-2.66208800
H	-0.56328800	-4.50822900	-3.48288000
H	1.50124200	-5.83768000	-3.88309200
C	3.66965400	2.31017300	2.18213500
C	5.00821900	2.74171600	2.22887100
C	3.02477000	2.00847400	3.39244700
C	5.67536800	2.87116500	3.44628900
H	5.52866300	2.98895800	1.30837200
C	3.69531000	2.13709100	4.61187900
H	1.99152000	1.67618000	3.39806300
C	5.02159900	2.56766400	4.64336700
H	6.70749800	3.21112000	3.45906400
H	3.17368800	1.90554300	5.53691200
H	5.54174400	2.66942200	5.59192900
C	3.09794100	3.65998400	-0.31378900
C	3.05581200	4.87284900	0.39345100
C	3.22852700	3.69217600	-1.70983900

C	3.14428600	6.08973200	-0.28257100
H	2.96016600	4.86827300	1.47559200
C	3.31161600	4.91186700	-2.38556700
H	3.27237700	2.75398200	-2.25670100
C	3.27181000	6.11149800	-1.67386800
H	3.11261300	7.02077100	0.27726000
H	3.41407000	4.92153800	-3.46747500
H	3.34109600	7.06049600	-2.19905900
Rh	3.42402900	0.13727100	-0.82798400
C	3.66457800	-0.63116200	-2.52290200
O	3.76432700	-0.93801900	-3.63535300
C	7.29722500	-1.44034100	-1.95021400
C	7.55678500	-2.75219300	-2.34850500
C	7.19678000	-3.82143000	-1.52387300
C	6.57826100	-3.56280600	-0.29913900
C	6.31804700	-2.24632500	0.09165700
C	6.66688300	-1.16391600	-0.72764300
H	7.58897800	-0.61573600	-2.59729500
H	8.04601300	-2.94000000	-3.30114300
H	7.40506200	-4.84369900	-1.82938600
H	6.30265800	-4.38444000	0.35793000
H	5.83635000	-2.05613900	1.04792700
C	6.39113100	0.26668400	-0.30694300
H	7.34257300	0.82542800	-0.35255600
C	5.34863300	1.01684100	-1.16175500
H	5.64419800	0.97831100	-2.21609900
H	5.35705800	2.07713400	-0.88856700
H	6.09124400	0.27856800	0.74903500

3:

C	-5.63383800	-2.14765100	2.72021900
C	-6.80454000	-1.73417700	2.02255200
C	-6.71882200	-0.62579900	1.13555100
C	-4.43696300	-1.49938300	2.54606700
C	-4.32290800	-0.39026100	1.65960800
C	-7.87717800	-0.18893200	0.42600800
C	-7.78948800	0.92637500	-0.46017700
C	-5.47413900	0.04961000	0.95457200
C	-5.38101900	1.16643400	0.06837200
C	-3.09087400	0.28544200	1.44447200
C	-2.98361100	1.35694900	0.58595800
C	-8.94472600	1.36249800	-1.16551700
C	-8.83364400	2.47990800	-2.04148700
C	-6.54492200	1.60458800	-0.63692300
C	-6.46649500	2.71780500	-1.51649500
C	-4.14180600	1.84407200	-0.11041600
C	-4.09814800	2.96440500	-0.99127900
C	-8.05864900	-2.39028900	2.18102900
C	-9.17208000	-1.97110300	1.49663900
C	-9.11625300	-0.86321800	0.60331100
C	-10.25556100	-0.40529600	-0.11782000
C	-10.17231800	0.66745800	-0.96991100
C	-7.63917100	3.13355000	-2.20977100
C	-5.21722300	3.38067100	-1.66728400
H	-10.12107900	-2.48471100	1.63076200
H	-11.20200500	-0.92276800	0.01912800
H	-7.57181100	3.98656300	-2.88057700
H	-5.15995100	4.24014000	-2.33083500

H	-2.21480100	-0.05959300	1.98744200
H	-3.55268500	-1.82894900	3.08633300
H	-5.70324300	-2.99390100	3.39940800
H	-8.12127800	-3.23726100	2.86001900
H	-11.05281400	1.00432400	-1.51175600
H	-9.71904700	2.81114300	-2.57878100
H	-3.16697700	3.50799600	-1.10118200
C	-1.67395300	2.09922700	0.45438000
O	-1.58792900	3.26824600	0.83046000
N	-0.59880700	1.44156500	-0.09635200
C	-0.64841600	0.09591500	-0.67562200
H	-1.68540200	-0.12231500	-0.93366000
H	-0.09464600	0.12525500	-1.61923200
C	0.64048800	2.21484900	-0.24479800
H	1.15210400	1.82113600	-1.12311500
H	0.35840600	3.24912500	-0.44766700
C	1.56702900	2.17469400	0.99506600
H	1.22754900	1.38766300	1.67357900
H	1.50277200	3.11523700	1.55152400
C	-0.10883000	-1.03668900	0.24487200
H	0.15133300	-0.61948700	1.22094400
H	-0.89435100	-1.77789800	0.42869400
P	3.34759700	1.74786300	0.58532100
P	1.44201600	-1.92219500	-0.35148300
C	1.42945200	-3.35599800	0.82554800
C	1.45923500	-3.10465000	2.20888400
C	1.45479200	-4.68980300	0.39011800
C	1.48447800	-4.15355500	3.12728700
H	1.47789300	-2.08118200	2.57451300
C	1.49023000	-5.74018600	1.31085400

H	1.43759000	-4.91371800	-0.67164000
C	1.49924300	-5.47719900	2.68084500
H	1.50160700	-3.93630900	4.19214400
H	1.50654800	-6.76617600	0.95228500
H	1.52403700	-6.29523800	3.39564600
C	0.85644500	-2.70704800	-1.92811500
C	1.78216700	-2.90592300	-2.96375700
C	-0.46939400	-3.13124000	-2.12346700
C	1.39498300	-3.50990700	-4.16241300
H	2.81029900	-2.58292700	-2.82744100
C	-0.85919900	-3.72525600	-3.32405200
H	-1.20736700	-3.00727600	-1.33636400
C	0.07305200	-3.91585400	-4.34706800
H	2.12628400	-3.65327300	-4.95334600
H	-1.89059200	-4.04008900	-3.45961100
H	-0.23159800	-4.37708100	-5.28275500
C	4.18669600	2.24212700	2.16586300
C	5.50139000	2.73876700	2.13845500
C	3.56995500	2.06247900	3.41459300
C	6.17466900	3.04746500	3.32045500
H	5.99808000	2.89923300	1.18565600
C	4.24256500	2.37570300	4.59737900
H	2.55662000	1.67744500	3.47608400
C	5.54777300	2.86720000	4.55557100
H	7.18897900	3.43558200	3.27519800
H	3.74255600	2.23566800	5.55233400
H	6.07130000	3.11018800	5.47620700
C	3.85836900	3.15920900	-0.50904600
C	3.39652600	4.47102900	-0.30687600
C	4.76341500	2.91992800	-1.55350500

C	3.81603300	5.50969600	-1.13755900
H	2.70961800	4.69081300	0.50536800
C	5.19336900	3.96261300	-2.37884600
H	5.13237300	1.91163500	-1.71931200
C	4.71628000	5.25750000	-2.17637300
H	3.44193900	6.51667700	-0.97204700
H	5.89445400	3.75770200	-3.18357800
H	5.04270800	6.06767400	-2.82310400
Rh	3.29305300	-0.44878300	-0.43074700
C	2.85619000	0.22871900	-2.16198100
O	2.72288200	0.53888400	-3.27215200
C	5.30348000	-2.72912600	-1.03192700
C	6.18181800	-2.80619800	-2.11116900
C	7.17218800	-1.83455600	-2.28354600
C	7.27454200	-0.78682400	-1.36449000
C	6.39611500	-0.70438700	-0.28409400
C	5.38640100	-1.67979000	-0.07905200
H	4.55936900	-3.50707100	-0.87992500
H	6.10283500	-3.63369300	-2.81218100
H	7.86346900	-1.89915400	-3.11968100
H	8.04855800	-0.03254200	-1.48493400
H	6.50341100	0.10338000	0.43306600
C	4.40798000	-1.59980000	1.02749400
H	3.90392500	-2.55727600	1.15935500
C	4.87929000	-1.08580600	2.37759300
H	5.57517100	-1.80248700	2.84269100
H	4.03459100	-0.96391800	3.06429000
H	5.38890600	-0.12197200	2.32546200

Supplementary References

1. Lang, R. et al. Hydroformylation of olefins by a rhodium single-atom catalyst with activity comparable to $RhCl(PPh_3)_3$. *Angew. Chem. Int. Ed.* **55**, 16054-16058 (2016).

2. Cai, Y. et al. Density Functional and Kinetic Monte Carlo Study of Cu-Catalyzed Cross-Dehydrogenative Coupling Reaction of Thiazoles with THF. *J. Org. Chem.* **81**, 1806−1812 (2016).

3. Frisch, M. J. et al. Gaussian 09, Revision D.01 (Gaussian, 2009).

4. Becke, A. Density-functional thermochemistry. III. The role of exact exchange. *J. Chem. Phys*. **98**, 5648 (1993).
